# Synthesis and Anti-Influenza A Virus Activity of 6′-amino-6′-deoxy-glucoglycerolipids Analogs

**DOI:** 10.3390/md14060116

**Published:** 2016-06-18

**Authors:** Li Ren, Jun Zhang, Haizhen Ma, Linlin Sun, Xiaoshuang Zhang, Guangli Yu, Huashi Guan, Wei Wang, Chunxia Li

**Affiliations:** Shandong Provincial Key Laboratory of Glycoscience and Glycoengineering, Key Laboratory of Marine Drugs of Ministry of Education, School of Medicine and Pharmacy, Ocean University of China, 5 Yushan Road, Qingdao 266003, China; renli@vip.163.com (L.R.); junerjasmine@163.com (J.Z.); zhangxtxm@163.com (H.M.); sunlinlinenjoylife@163.com (L.S.); xiaoshuangzhang89@126.com (X.Z.); glyu@ouc.edu.cn (G.Y.); hsguan@ouc.edu.cn (H.G.)

**Keywords:** synthesis, aminoglucoglycerolipids, anti-influenza A virus

## Abstract

A series of aminoglucoglycerolipids derivatives had been synthesized, including 6′-acylamido-glucoglycerolipids **1a**–**1f** and corresponding 2′-acylamido-glucoglycerolipids **2a**–**2c** bearing different fatty acids, glucosyl diglycerides **3a**–**3e** bearing different functional groups at C-6′ and ether-linked glucoglycerolipids **4a**–**4c** with double-tailed alkyl alcohol. The anti-influenza A virus (IAV) activity was evaluated by the cytopathic effects (CPE) inhibition assay. The results indicated that the integral structure of the aminoglycoglycerolipid was essential for the inhibition of IAV in MDCK cells. Furthermore, oral administration of compound **1d** was able to significantly improve survival and decrease pulmonary viral titers in IAV-infected mice, which suggested that compound **1d** merited further investigation as a novel anti-IAV candidate in the future.

## 1. Introduction

Type A influenza, as an infectious disease caused by influenza A virus (IAV), once inflicted more casualties than any other infectious diseases in Europe. It had been the cause of at least three pandemics in the last century, the most severe leading to more than 40 million fatalities in 1918–1919 [1]. Moreover, the 2009 outbreak of swine-origin influenza A/H1N1 [2,3] continued to affect many countries, and caused over 18,000 deaths worldwide [4]. Considering the frequency of influenza pandemics, it is urgent to develop novel anti-IAV drugs with high efficiency.

**Figure 1:**
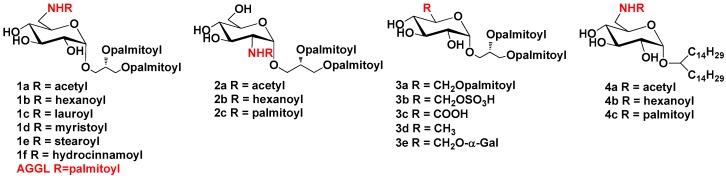
Structures of the **AGGL** and synthesized compounds **1**–**4**.

Glycoglycerolipids occur widely in marine algae [5,6,7,8], cyanobacteria [9,10,11] and higher plants. The natural glycoglycerolipids possess various biological activities, such as anti-tumor [12,13], anti-viral [14,15,16,17], and anti-inflammatory activities [18], which make them valuable molecular targets for further investigation [19]. In our previous work [20,21,22] synthetic aminoglycoglycerolipids derived from marine natural product 1,2-dipalmitoyl-3-(*N*-palmitoyl-6′-amino-6′-deoxy-α-d-glucosyl)-*sn*-glycerol (AGGL, Figure 1) [23] were found to possess notable anti-IAV activity. Moreover, preliminary results indicated that the type and the length of the acids in the aminomannosylglycerols could influence the inhibitory effect [20]. To explore more potent anti-IAV leading drug candidates, herein, we synthesized new series of aminoglycoglycerolipid derivatives and evaluated their anti-IAV activity in MDCK cells. Some compounds displayed potent antiviral effects, and the primary structure-activity relationship was summarized. Compound **1d** was further found to be able to improve survival and decrease pulmonary viral titers in IAV-infected mice significantly.

## 2. Results and Discussion

### 2.1. Chemistry

New series of **AGGL** derivatives **1**–**4** were designed as Figure 1. The structural modification of **AGGL** focused on the fatty acid amide linked at C-6′position of the sugar ring and the glyceride attached to anomeric carbon, including 6′-acylamido-glucoglycerolipids **1a**–**1f** and corresponding 2′-acylamido-glucoglycerolipids **2a**–**2c** bearing different fatty acids, glucosyl diglycerides **3a**–**3e** bearing different functional groups at C-6′ and ether-linked glucoglycerolipids **4a**–**4c** with double-tailed alkyl alcohol. The synthetic routes were described as follows.

#### 2.1.1. Synthesis of Compounds **1a**–**1f**

**Scheme 1:**
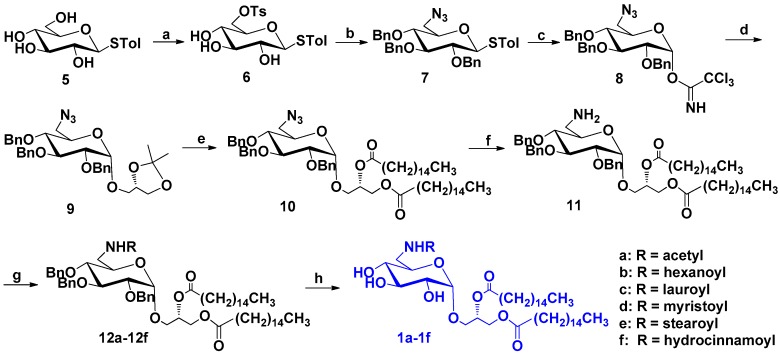
Reagents and conditions: (**a**) TsCl, DMAP, py, 79%; (**b**) (i) NaN_3_, DMF; (ii) BnBr, NaH, DMF, 86% for 2 steps; (**c**) (i) NBS, CO(CH_3_)_2_/H_2_O; (ii) CNCCl_3_, DBU, CH_2_Cl_2_, 89% for 2 steps; (**d**) (*S*)-isopropylideneglycerol, TMSOTf, Et_2_O, 97%; (**e**) (i) *p*-TsOH, MeOH, 89%; (ii) CH_3_(CH_2_)_14_COCl, DMAP, py, 96%; (**f**) 10% Pd/C, H_2_, AcOEt/MeOH, 95%; (**g**) (i) EDCI, HOBt, ROH, CH_2_Cl_2_ or Ac_2_O, CH_2_Cl_2_, 41%–89%; (**h**) 20% Pd(OH)_2_/C, H_2_, THF/*i*-PrOH, 83%–96%.

6′-amide-6′-deoxy-α-d-glucoglycerolipids **1a**–**1f** bearing different fatty acids at amino group were synthesized as illustrated in Scheme 1. Activation of thioglycoside **5** [24] with *p*-toluene sulfonyl chloride, followed by treatment **6** with sodium azide and benzyl bromide in one-pot method gave compound **7**. The *p*-tolylthio group of **7** was removed by *N*-bromosuccinimide (NBS) in acetone-water, then treatment with trichloroacetonitrile (CCl_3_CN) and 1,8-diazabicyclo[5.4.0]-undec-7-ene (DBU) afforded trichloroacetimidate **8** in 89% as the glycosyl donor. Glycosylation of (*S*)-isopropylideneglycerol with donor **8** under trimethylsilyl trifluoromethanesulfonate (TMSOTf) activation in absolute ether afforded **9** as an inseparable anomeric mixture (α/β = 12:1) in 97% yield. The small amount of β-anomer can be removed by silica gel column chromatography in the following step of hydrolysis with TsOH in methanol (89%). Treatment the α-anomer diol with excess palmitoyl chloride in the presence of *N*,*N*-dimethylaminopyridine (DMAP) produced **10** in 96% yield. Reduction of the azido group by Pd/H_2_ yielded the amino derivative **11** in 95% yield. Introduction of acetyl group on C6′–NH_2_ with Ac_2_O and C6-C16 fatty acids under the condition of EDCI/HOBt in CH_2_Cl_2_ afforded **12a**–**12f**. Then final compounds **1a**–**1f** were obtained by removal of the benzyl with H_2_/Pd(OH)_2_ in 83%–96% yields.

#### 2.1.2. Synthesis of Compounds **2a**–**2c**

**Scheme 2:**
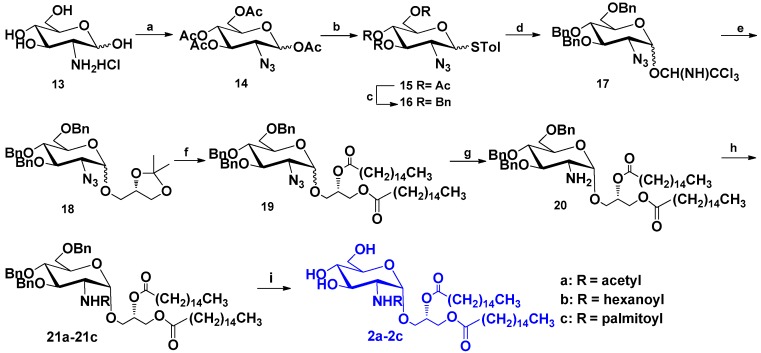
Reagents and conditions: (**a**) (i) Imidazole-1-sulfonyl azide hydrochloride, CuSO_4_, K_2_CO_3_, MeOH; (ii) Ac_2_O, py, 81% for 2 steps; (**b**) TolSH, BF_3_·Et_2_O, CH_2_Cl_2_, 85%; (**c**) (i) CH_3_ONa, CH_3_OH; (ii) BnBr, NaH, DMF, 98% for 2 steps; (**d**) (i) NBS, (CH_3_)_2_CO/H_2_O; (ii) CNCCl_3_, DBU, CH_2_Cl_2_, 82% for 2 steps; (**e**) (*S*)-isopropylideneglycerol, TMSOTf, Et_2_O, 93%; (**f**) (i) *p*-TsOH, MeOH; (ii) CH_3_(CH_2_)_14_COCl, DMAP, py, 82% for 2 steps; (**g**) 10% Pd/C, H_2_, AcOEt/MeOH, 54%; (**h**) (i) Ac_2_O, CH_2_Cl_2_ or ROH, EDCI, HOBt, CH_2_Cl_2_, 64%–96%; (ii) 20% Pd(OH)_2_/C, H_2_, THF/MeOH, 84%–96%. (**i**).

In addition to the 6′-amides **1a**–**1f**, we also synthesized the related 2′-amide glycoglycerolipids **2a**–**2c** in order to compare the influence on biological activity of the position of amide group. As shown in Scheme 2, the synthesis started with the diazotransfer reaction [25] to introduce an azide group into 2-C of the glucosamine (**13**), followed by acetylation in one-pot method to give **14** in 81% yield. The acetate glycoside **14** reacted with *p*-toluene thiophenol to afford thioglycoside **15** in 85% yield. The acetyl protective group of **15** was changed by benzyl groups to give **16** in 98% yield, and then the *p*-tolylthio group was transformed to trichloroacetimidate to yield donor **17** (82%). Glycosylation of **17** with (*S*)-isopropylideneglycerol under TMSOTf condition afforded glycolipid anomers **18** (α/β = 2:1). After hydrolyzation, esterification and reduction according to the above procedures of **11**, α-anomer **20** (54%) was producecd and isolated by silica gel column chromatography. Acylation of the resulting C2′-NH_2_ followed by hydrogenation with H_2_/Pd(OH)_2_ generated the target compounds **2a**–**2c**.

#### 2.1.3. Synthesis of Compounds **3a**–**3e**

**Scheme 3:**
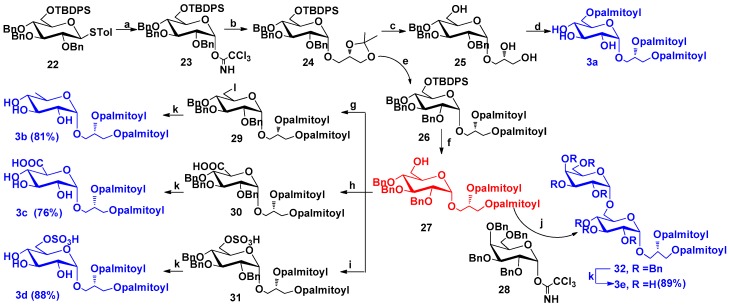
Reagents and conditions: (**a**) (i) NBS, (CH_3_O)_2_CO/H_2_O, 90%; (ii) CNCCl_3_, DBU, CH_2_Cl_2_, 92%; (**b**) (*S*)-isopropylideneglycerol, TMSOTf, Et_2_O, 83%; (**c**) *p*-TsOH, MeOH, 82%; (**d**) (i) CH_3_(CH_2_)_14_COCl, DMAP, py; (ii) 20% Pd(OH)_2_/C, H_2_, AcOEt/MeOH, 62% for 2 steps; (**e**) (i) CSA, MeOH, 93%; (ii) CH_3_(CH_2_)_14_COCl, DMAP, py, 88%; (**f**) TBAF, THF, 84%; (**g**) PPh_3_, I_2_, imidazole, toluene, 70%; (**h**) TEMPO, BAIB, CH_2_Cl_2_/H_2_O, 92%; (**i**) SO_3_·py, DMF, 86%; (**j**) **28**, TMSOTf, Et_2_O, 75%; (**k**) 20% Pd(OH)_2_/C, H_2_, AcOEt/MeOH.

Glucosyl diglycerides **3a**–**3e** bearing different functional groups at C-6′ were synthesized as illustrated in Scheme 3. Starting from the synthetic compound **22** [26], the trichloroacetimidate donor **23** was prepared, followed by glycosylation with (*S*)-isopropylideneglycerol to afford the key intermediate **24**. TBDPS was chosen to selectively protect the primary hydroxyl of the glucose, which might also provide a remote participating effect in the latter glycosylation reaction to afford absolute α-linked glycoside **24** [27,28]. Under the condition of *p*-TsOH, acetonide and the TBDPS groups in **24** were removed simultaneously (**25**, 82%). After esterification and deprotection, **3a** was obtained in 62% yield. Besides, in the catalyst of camphorsulfonic acid (CSA), the acetonide of **24** could be selectively hydrolyzed, then esterification of the diols and removal of the silyl ether group to give **27** in 84% yield. Compound **27** was first converted to the corresponding iodo analog **29** upon treatment with triphenylphosphine, imidazole and iodine [29]. The glucuronic acid derivative **30** was prepared upon oxidation of the 6′-OH of **27** into the corresponding carboxylic acid via a TEMPO/BAIB oxidation [30]. 6′-*O*-sulfated derivative **31** was prepared under the treatment with Py·SO_3_ complex in DMF at room temperature [31]. In addition, coupling of **27** with galacosyl trichloroacetimidate donor **28** [32] using a catalytic amount of TMSOTf in Et_2_O gave the desired α-(1→6)-linked disaccharide **32** in 75% yield. Deprotection of **29**–**32** by Pd-catalyzed hydrogenation afforded the desired compounds **3b**–**3e**.

#### 2.1.4. Synthesis of Compounds **4a**–**4c**

**Scheme 4:**
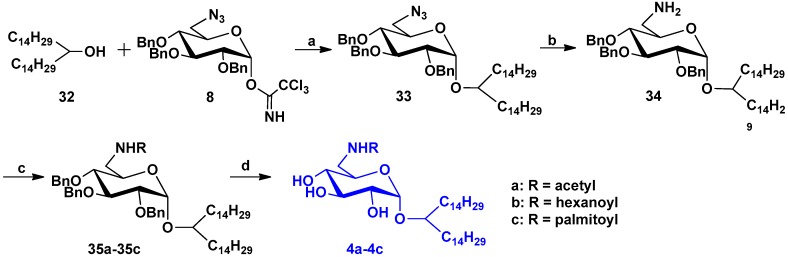
Reagents and conditions: (**a**) TMSOTf, Et_2_O, 49%; (**b**) 10% Pd/C, H_2_, AcOEt/MeOH, 88%; (**c**) Ac_2_O, CH_2_Cl_2_ or ROH, EDCI, HOBt, CH_2_Cl_2_, 58%–87%; (**d**) 20% Pd(OH)_2_/C, H_2_, THF/MeOH, 89%–96%.

Diacylglycerol residue was substituted by double-tailed alcohol linked at the anomeric carbon of the 6′-amide-6′-deoxy-α-d-glucose to afford the derivatives **4a**–**4c**. As shown in Scheme 4, trichloroacetimidate donor **8** reacted with bis (tetradecyl) carbinol **32** [33] under TMSOTf activation in Et_2_O to afford the absolute α-linked intermediate **33** in 49% yield. After reduction, acylation and deprotection, target analogs **4a**–**4c** were obtained.

### 2.2. Biological Evaluation

#### 2.2.1. Inhibition of Influenza A Virus Multiplication *in Vitro*

6′-amino-6′-deoxy-glycoglycerolipid (**AGGL**) and its derivatives were tested for their antiviral activity by the cytopathic effects (CPE) inhibition assay [34]. As shown in Table 1, natural **AGGL** displayed moderate virus inhibition at 50 μM. Compounds **1b** and **1d**, which contained saturated fatty acyl chains of C6 and C14 at 6′-NH_2_, displayed notable virus inhibition among the aminoglucoglycerolipids bearing linear fatty acids with C2–C18 length (**1a**–**e**), suggesting that the optimum length of saturated fatty acyl chains may be important for their anti-IAV effects *in vitro*. Compound **2b** bearing hexanoic acid (C6) at 2′-NH_2_ also exhibited similar inhibitory activity with the control of ribavirin. Whereas, compound **1b** which also had a hexanoyl group at a different position, showed comparably good anti-IAV activity (46.1%) *in vitro*, suggesting that the hexanoyl group may be indispensable for rational design of novel anti-IAV drugs based on AGGL structure. In addition, compound **1f** with an aromatic ring at the acylamino group displayed a decreased effect on inhibition of IAV multiplication in MDCK cells, which was consistent with our previous results [20]. Moreover, our previous result [20] indicated that the tripalmitoyl derivative of the 6-aminomannoglycerolipid had 48% IAV inhibition at 50 μM, which was more than that of AGGL (25.5%) at the same concentration, suggesting that mannose may be able to increase the activity of these lipids. In addition, compounds **3a**–**3d** and **4a**–**4c**, which replaced the fatty acyl amide with other groups (**3a**–**3d**), or substituted the glycerolipid with a dialkyl alcohol (**4a**–**4c**), had much lower inhibition effect. These results indicated that the acylamino and glycerol groups of the glycolipids were essential for the inhibition of IAV multiplication. Surprisingly, glucosyl diglyceride **3e** bearing another galactose at C-6′ exerted excellent inhibitory effect (51.5% at 50 μM), and hence the introduction of galactose at 6′-position of the glucosyl moiety may be able to enhance its activity.

#### 2.2.2. IAV Infection *in Vivo*

**Figure 2:**
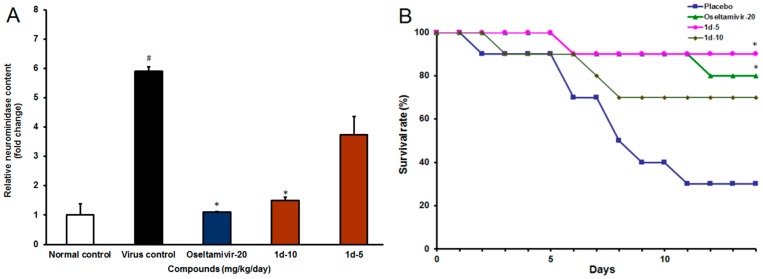
The therapeutic effect of **1d** on influenza A virus-infected mice. (**A**) Viral titers in lungs evaluated by performing neuraminidase activity assay. The mean fluorescence intensity of each sample was used to evaluate the relative neuraminidase content. The intensity of normal control group was assigned values of 1.0 and the data presented as mean ± S.D. (*n* = 3). Significance: # *p* < 0.05 *vs.* normal control group; * *p* < 0.05 *vs.* virus control group (placebo); (**B**) Survival rate. IAV infected mice received intraperitoneal therapy once daily with drugs or placebo for the entire experiment. Results are expressed as percentage of survival, evaluated daily for 15 d. Significance: * *p* < 0.05 *vs.* control group (placebo).

Considering the structure characteristic and bioactivity *in vitro*, potent compound **1d** (IC_50_ = 60.8 μM, close to that of Ribavirin (IC_50_ = 49.7 μM)) in the preliminary anti-viral screening was selected for further study of its inhibitory effect on IAV infection *in vivo*. In brief, IAV-infected mice received oral administration of Oseltamivir (20 mg/kg/d), compound **1d** (5, 10 mg/kg/d) or placebo (PBS) once daily for the entire experiment, and then sacrificed at 4 d p.i. Subsequently, the pulmonary viral titers were determined by performing neuraminidase activity assay [35,36]. As shown in Table 2, compound **1d** could reduce the lung index in IAV-infected mice with an inhibition rate of 15.3% at a dose of 5 mg/kg/d. Moreover, neuraminidase (NA) assay exhibited a significant reduction of virus titers in the lungs of **1d**-treated mice (10 mg/kg/d) 4 d post infection, as compared to the control group (*p* < 0.05) (Figure 2A). The data suggested that oral therapy of infected mice with **1d** resulted in a reduction of viral titers in the lung [37].

Furthermore, influenza A virus can induce lethal infections in certain mouse strains usually within 2 weeks [38]. Therefore, the survival experiments were also performed to evaluate the effect of compound **1d** on the survival of IAV-infected mice. As shown in Figure 2B, the survival rate significantly increased in the **1d** and oseltamivir-treated groups, as compared to the placebo-treated control group (*p* < 0.5). By d 14 after infection, only 30% of the individuals in the placebo group survived whereas 90% of those in the **1d** (5 mg/kg/d) treated group survived, superior to that in the Oseltamivir treated group (Figure 2B).

Moreover, some researchers reported that small molecules such as flavonoids had no inhibitory effect against inflammatory related diseases at excessive concentrations, and the potential toxicity of flavonoids at high doses was possibly due to the generation of reactive oxygen species [39]. Herein, the anti-IAV effect of compound **1d** also declined when elevating the dose from 5 mg/kg/d to 10 mg/kg/d, thus we supposed that treatment of compound **1d** at high dose (10 mg/kg/d) may cause toxicity to mice due to the generation of reactive oxygen species or excessive pro-inflammatory cytokines in IAV infected mice. In addition, Sugawara *et al.* reported that oral administration of glycoglycerolipids may cause hydrolytic degradation and poor adsorption of these compounds [40]. However, Maeda *et al.* showed that the oral administration of cyclodextrin-galactosyldiacylglycerol complex could inhibit the tumor growth in mice despite the probable digestive degradation of these compounds [41]. Thus, the oral administration of compound **1d** may also cause hydrolytic degradation but it may be able to inhibit IAV replication *in vivo* through other mechanisms.

## 3. Experimental Section

### 3.1. Chemical Procedures

#### 3.1.1. General Information

Solvents were purified in a conventional manner. Thin layer chromatography (TLC) was performed on precoated HSGF254 plates (Yantai Chemical Industry Institute, Yantai, China). Flash column chromatography was performed on silica gel (200–300 mesh, Qingdao Haiyang Chemical Co., Ltd., Qingdao, China). ^1^H-NMR and ^13^C-NMR spectra were taken on a JEOL JNM-ECP 600 MHz (JEOL Ltd., Tokyo, Japan) and an Agilent 500 MHz DD2 spectrometer (Agilent Technologies, Santa Clara, CA, USA) with tetramethylsilane (Me4Si) as the internal standard, and chemical shifts were recorded as δ values. Mass spectra were recorded on a Global Q-TOF mass spectrometer (Waters Ltd., Wilmslow, UK) and IonSpec 4.7 Tesla FTMS (Varian Inc., Palo Alto, CA, USA) (MALDI/DHB).

#### 3.1.2. Chemistry: General Methods

##### General Procedure for Compounds **1a–1f**

A solution of **12a**–**12f** in THF/*i*-PrOH (9:1) was treated with 20% palladium hydroxide and stirred at ambient temperature under hydrogen atmosphere for 3.5 h. After filtration, the solvent was evaporated and the residue was purified by silica column chromatography (CH_2_Cl_2_-MeOH 30:1) to afford **1a**–**1f** (83%–96%) as white solids.

##### 1,2-Dipalmitoyl-3-*O*-(*N*-acetyl-6′-amino-6′-deoxy-α-d-glucopyranosyl)-*sn*-glycerol (**1a**)

30 mg, 91% yield; ^1^H-NMR (600 MHz, CDCl_3_): δ 6.26–6.18 (m, 1H, N***H***-CO), 5.24–5.20 (m, 1H, ***H_sn-2_***), 4.80 (d, *J* = 3.5 Hz, 1H, ***H-1)***, 4.38 (dd, *J* = 12.0, 3.6 Hz, 1H, ***H_sn-1a_***), 4.14 (dd, *J* = 12.0, 6.1 Hz, 1H, ***H_sn-1b_***), 3.93–3.87 (m, 1H, ***H-5***), 3.77 (dd, *J* = 10.8, 5.1 Hz, 1H, ***H_sn-3a_***), 3.73 (t, *J* = 9.0 Hz, 1H, ***H-3***), 3.62 (dd, *J* = 10.9, 5.8Hz, 1H, ***H_sn-3b_***), 3.58–3.55 (m, 1H, ***H-6a***), 3.52–3.48 (m, 1H, ***H-2***), 3.18–3.04 (m, 2H, ***H-4***, ***H-6b***), 2.32–2.28 (m, 4H, 2 × CO–C***H_2_***), 2.04 (s, 3H, NH–CO–C***H_3_***), 1.63–1.58 (m, 4H, 2 × CO–CH_2_–C***H_2_)***, 1.29–1.24 (m, 48H, 2 × CH_2_–CH_2_–(C***H_2_***)_12_–CH_3_), 0.87 (t, *J* = 7.0 Hz, 6H, 2 × C***H_3_***); ^13^C-NMR (150 MHz, CDCl_3_): δ 172.8, 172.5, 171.7, 98.7, 72.4, 71.5, 70.3, 69.5, 69.1, 65.9, 61.6, 39.2, 33.5, 33.4, 31.2, 29.0–28.4, 24.2, 22.3, 22.0, 13.4; HR-ESI-MS *m*/*z* calcd. for C_43_H_82_NO_10_ [M + H]^+^ 772.5933, found 772.5944.

##### 1,2-Dipalmitoyl-3-*O*-(*N*-hexanoyl-6′-amino-6′-deoxy-α-d-glucopyranosyl)-*sn*-glycerol (**1b**)

44 mg, 90% yield; ^1^H-NMR (600 MHz, CDCl_3_): δ 5.96 (dd, *J* = 8.0, 5.1 Hz, 1H, N***H***–CO), 5.25–5.21 (m, 1H, ***H_sn-2_***), 4.80 (d, *J* = 4.0 Hz, 1H, ***H-1)***, 4.38 (dd, *J* = 12.0, 4.0 Hz, 1H, ***H_sn-1a_***), 4.14 (dd, *J* = 12.0, 6.0 Hz, 1H, ***H_sn-1b_***), 4.01–3.97 (m, 1H, ***H-5***), 3.78 (dd, *J* = 11.0, 4.8 Hz, 1H, ***H_sn-3a_***), 3.73 (t, *J* = 9.3 Hz, 1H, ***H-3***), 3.62 (dd, *J* = 11.0, 6.0 Hz, 1H, ***H_sn-3b_***), 3.57 (dt, *J* = 9.7, 2.3 Hz, 1H, ***H-6a***), 3.54–3.46 (m, 1H, ***H-2***), 3.12–3.04 (m, 2H, ***H-4***, ***H-6b***), 2.32–2.28 (m, 4H, 2 × CO–C***H_2_*)**, 2.24 (dt, *J* = 7.4, 3.1 Hz, 2H, NH–CO–C***H_2_***), 1.63–1.58 (m, 6H, 3 × CO–CH_2_–C***H_2_***), 1.29–1.24 (m, 52H, 2 × CH_2_–CH_2_–(C***H_2_***)_12_–CH_3_, CH_2_–CH_2_–(C***H_2_***)_2_–CH_3_), 0.91–0.84 (m, 9H, 3 × C***H_3_***); ^13^C-NMR (150 MHz, CDCl_3_): δ 175.0, 172.7, 172.5, 98.7, 72.3, 71.6, 70.4, 69.2, 69.1, 66.1, 61.4, 39.0, 35.7, 33.5, 33.3, 31.2, 30.7, 29.0–28.4, 24.6, 24.1, 21.9, 21.6, 13.4, 13.2; HR-ESI-MS *m*/*z* calcd. for C_47_H_90_NO_10_ [M + H]^+^ 828.6559, found 828.6557.

##### 1,2-Dipalmitoyl-3-*O*-(*N*-lauroyl-6′-amino-6′-deoxy-α-d-glucopyranosyl)-*sn*-glycerol (**1c**)

48 mg, 89% yield; ^1^H-NMR (600 MHz, CDCl_3_): δ 5.91 (dd, *J* = 8.1, 4.7 Hz, 1H, N***H***–CO), 5.24–5.20 (m, 1H, ***H_sn-2_***), 4.80 (d, *J* = 3.9 Hz, 1H, ***H-1)***, 4.38 (dd, *J* = 11.9, 4.0 Hz, 1H, ***H_sn-1a_***), 4.12 (dd, *J* = 11.9, 5.9 Hz, 1H, ***H_sn-1b_***), 4.01 (ddd, *J* = 15.0, 8.3, 2.4 Hz, 1H, ***H-5***), 3.78 (dd, *J* = 11.0, 4.8 Hz, 1H, ***H_sn-3a_***), 3.73 (t, *J* = 9.3 Hz, 1H, ***H-3***), 3.62 (dd, *J* = 11.0, 6.0 Hz, 1H, ***H_sn-3b_***), 3.57 (dt, *J* = 9.6, 2.5 Hz, 1H, ***H-6a***), 3.52–3.48 (m, 1H, ***H-2***), 3.09 (t, *J* = 9.6 Hz, 1H, ***H-4***), 3.04 (ddd, *J* = 14.7, 4.5, 2.8 Hz, 1H, ***H-6b***), 2.32–2.28 (m, 4H, 2 × CO–C***H_2_***), 2.25–2.22 (m, 2H, NH–CO–C***H_2_***), 1.63–1.58 (m, 6H, 3 × CO–CH_2_–C***H_2_***), 1.29–1.24 (m, 64H, 2 × CH_2_–CH_2_–(C***H_2_***)_12_–CH_3_, CH_2_–CH_2_–(C***H_2_***)_8_–CH_3_), 0.87 (t, *J* = 7.0 Hz, 9H, 3 × C***H_3_***); ^13^C-NMR (150 MHz, CDCl_3_): δ 175.0, 172.7, 172.5, 98.7, 72.3, 71.6, 70.4, 69.2, 69.1, 66.1, 61.3, 38.9, 35.7, 33.5, 33.3, 31.2, 29.0–28.4, 24.9, 24.1, 21.9, 13.3; HR-ESI-MS *m*/*z* calcd. for C_53_H_102_NO_10_ [M + H]^+^ 912.7498, found 912.7493.

##### 1,2-Dipalmitoyl-3-*O*-(*N*-myristoyl-6′-amino-6′-deoxy-α-d-glucopyranosyl)-*sn*-glycerol (**1d**)

45 mg, 83% yield; ^1^H-NMR (600 MHz, CDCl_3_): δ 5.95 (dd, *J* = 7.9, 4.9 Hz, 1H, N***H***-CO), 5.24–5.20 (m, 1H, ***H_sn-2_***), 4.80 (d, *J* = 3.9 Hz, 1H, ***H-1***), 4.38 (dd, *J* = 11.9, 4.0 Hz, 1H, ***H_sn-1a_***), 4.12 (dd, *J* = 12.0, 6.0 Hz, 1H, ***H_sn-1b_***), 3.99 (ddd, *J* = 14.9, 8.3, 2.7 Hz, 1H, ***H-5***), 3.77 (dd, *J* = 11.0, 4.8 Hz, 1H, ***H_sn-3a_***), 3.73 (t, *J* = 9.3 Hz, 1H, ***H-3***), 3.62 (dd, *J* = 11.0, 5.9 Hz, 1H, ***H_sn-3b_***), 3.57 (dt, *J* = 9.8, 2.7 Hz, 1H, ***H-6a***), 3.48 (dd, *J* = 9.6, 3.9 Hz, 1H, ***H-2***), 3.10 (t, *J* = 9.5 Hz, 1H, ***H-4***), 3.06–3.03 (m, 1H, ***H-6b***), 2.33–2.28 (m, 4H, 2 × CO–C***H_2_***), 2.26–2.17 (m, 2H, NH–CO–C***H_2_***), 1.62–1.58 (m, 6H, 3 × CO–CH_2_–C***H_2_***), 1.29–1.24 (m, 68H, 2 × CH_2_–CH_2_–(C***H_2_***)_12_–CH_3_, CH_2_–CH_2_–(C***H_2_***)_10_–CH_3_), 0.87 (t, *J* = 7.0 Hz, 9H, 3 × C***H_3_***); ^13^C-NMR (150 MHz, CDCl_3_): δ 175.0, 172.8, 172.5, 98.7, 72.4, 71.6, 70.4, 69.4, 69.2, 66.1, 61.5, 39.0, 35.8, 33.6, 33.4, 31.2, 29.1–28.4, 25.0, 24.2, 22.0, 13.4; HR-ESI-MS *m*/*z* calcd. for C_55_H_106_NO_10_ [M + H]^+^ 940.7811, found 940.7801.

##### 1,2-Dipalmitoyl-3-*O*-(*N*-stearoyl-6′-amino-6′-deoxy-α-d-glucopyranosyl)-*sn*-glycerol (1**e**)

68 mg, 96% yield; 1H-NMR (600 MHz, CDCl3): δ 6.03–5.99 (m, 1H, NH-CO), 5.25–5.20 (m, 1H, Hsn-2), 4.80 (d, *J* = 4.0 Hz, 1H, ***H-1***), 4.38 (dd, *J* = 11.9, 3.9 Hz, 1H, ***H_sn-1a_***), 4.13 (dd, *J* = 11.9, 6.0 Hz, 1H, ***H_sn-1b_***), 3.98–3.95 (m, 1H, ***H-5***), 3.77 (dd, *J* = 10.9, 4.9 Hz, 1H, ***H_sn-3a_***), 3.73 (t, *J* = 9.0 Hz, 1H, ***H-3***), 3.62(dd, *J* = 11.0, 5.9 Hz, 1H, ***H_sn-3b_***), 3.57 (dt, 1H, *J* = 9.2, 2.8 Hz, ***H-6a***), 3.47 (dd, *J* = 9.2, 2.9 Hz, 1H, ***H-2***), 3.13–3.05 (m, 2H, ***H-4***, ***H-6b***), 2.32–2.27 (m, 4H, 2 × CO–C***H_2_***), 2.26–2.19 (m, 2H, NH–CO–C***H_2_***), 1.63–1.56 (m, 6H, 3 × CO–CH_2_–C***H_2_***), 1.33–1.22 (m, 76H, 2 × CH_2_–CH_2_–(C***H_2_***)_12_–CH_3_, CH_2_–CH_2_–(C***H_2_***)_14_–CH_3_), 0.87 (t, *J* = 7.0 Hz, 9H, 3 × C***H_3_***); ^13^C-NMR (150 MHz, CDCl_3_): δ 174.9, 172.8, 172.6, 98.7, 72.4, 71.6, 70.4, 69.5, 69.2, 66.1, 61.6, 39.1, 35.8, 33.6, 33.4, 31.2, 29.0–28.4, 25.0, 24.2, 22.0, 13.4; HR-ESI-MS *m*/*z* calcd. for C_59_H_114_NO_10_ [M + H]^+^ 996.8437, found 996.8425.

##### 1,2-Dipalmitoyl-3-*O*-(*N*-hydrocinnamoyl-6′-amino-6′-deoxy-α-d-glucopyranosyl)-*sn*-glycerol (**1f**)

30 mg, 88% yield; ^1^H-NMR (600 MHz, CDCl_3_): δ 7.30 (t, *J* = 7.5 Hz, 2H, Ar***H***), 7.23–7.18 (m, 3H, Ar***H***), 5.86 (dd, *J* = 8.1, 4.6 Hz, 1H, N***H***–CO), 5.22–5.18 (m, 1H, ***H_sn-2_***), 4.73 (d, *J* = 3.9 Hz, 1H, ***H-1)***, 4.36 (dd, *J* = 12.0, 4.0 Hz, 1H, ***H_sn-1a_***), 4.10 (dd, *J* = 12.0, 6.0 Hz, 1H, ***H_sn-1b_***), 3.99–3.96 (m, 1H, ***H-5***), 3.74 (dd, *J* = 11.0, 4.7 Hz, 1H, ***H_sn-3a_***), 3.69 (t, *J* = 9.4 Hz, 1H, ***H-3***), 3.59 (dd, *J* = 12.0, 6.0 Hz, 1H, ***H_sn-3b_***), 3.51 (dt, *J* = 9.9, 2.5 Hz, 1H, ***H-6a***), 3.02–2.91 (m, 4H, ***H-2***, ***H-6b***, NHCO-C***H_2_***), 2.90 (t, *J* = 9.4 Hz, 1H, ***H-4***), 2.58–2.54 (m, 2H, C***H_2_***Ph), 2.29–2.25 (m, 4H, 2 × CO–C***H_2_***), 1.60–1.57 (m, 4H, 2 × CO–CH_2_–C***H_2_***), 1.29–1.24 (m, 48H, 2 × CH_2_–CH_2_–(C***H_2_***)_12_–CH_3_), 0.87 (t, *J* = 7.0 Hz, 6H, 2 × C***H_3_***); ^13^C-NMR (150 MHz, CDCl_3_): δ 173.8, 172.9, 172.6, 139.7, 128.1, 127.7, 125.8, 98.7, 72.4, 71.6, 70.3, 69.2, 69.1, 66.1, 61.6, 39.1, 37.5, 33.6, 33.5, 31.3, 30.8, 29.4–28.5, 24.2, 22.1, 13.5; HR-ESI-MS *m*/*z* calcd. for C_50_H_88_NO_10_ [M + H]^+^ 862.6403, found 862.6394.

##### General Procedure for **2a**–**2c**

Pd(OH)_2_/C (20%) was added to a mixture of compounds **21a**–**21c** in THF/MeOH (1:1). Upon stirring for 4 h under H_2_, the mixture was filtered off and the solvent was evaporated. After purification by column chromatography (CH_2_Cl_2_/MeOH 20:1), compounds **2a**–**2c** (84%–96%) were afforded as white solids.

##### 1,2-Dipalmitoyl-3-*O*-(*N*-acetyl-2′-amino-2′-deoxy-α-d-glucopyranosyl)-*sn*-glycerol (**2a**)

40 mg, 92% yield; ^1^H-NMR (600 MHz, CDCl_3_): δ 6.57 (d, *J* = 8.9 Hz, 1H, N***H***CO), 5.20–5.18 (m, 1H, ***H_sn-2_***), 4.77 (d, *J* = 3.3 Hz, 1H, ***H-1***), 4.46 (dd, *J* = 11.9, 4.1 Hz, 1H, ***H_sn-1a_***), 4.08–4.06 (m, 1H, ***H-2***), 4.05 (dd, *J* = 11.9, 6.0 Hz, 1H, ***H_sn-1b_***), 3.91–3.88 (m, 1H, ***H-6a***), 3.79–3.76 (m, 1H, ***H-6b***), 3.75 (dd, *J* = 11.2, 4.9 Hz, 1H, ***H_sn-3a_***), 3.69–3.67 (m, 2H, ***H-5***, ***H-3***), 3.57–3.55 (m, 1H, ***H-4***), 3.52 (dd, *J* = 11.0, 5.7 Hz, 1H, ***H_sn-3b_***), 2.32–2.28 (m, 4H, 2 × CO–C***H_2_***), 2.05 (s, 3H, NH–CO–C***H_3_***), 1.61–1.58 (m, 4H, 2 × CO–CH_2_–C***H_2_***), 1.29–1.24 (m, 48H, 2 × CH_2_–CH_2_–(C***H_2_***)_12_–CH_3_), 0.87 (t, *J* = 7.0 Hz, 6H, 2 × C***H_3_***); ^13^C-NMR (126 MHz, CDCl_3_): δ 173.56, 173.18, 172.40, 97.93, 73.14, 72.01, 70.33, 69.83, 65.66, 61.94, 61.31, 53.52, 34.28, 34.10, 31.92, 29.71–29.13, 24.92, 24.87, 23.09, 22.69, 14.12; HR-ESI-MS *m*/*z* calcd. for C_43_H_82_NO_10_ [M + H]^+^ 772.5933, found 772.5925.

##### 1,2-Dipalmitoyl-3-*O*-(*N*-hexanoyl-2′-amino-2′-deoxy-α-d-glucopyranosyl)-*sn*-glycerol (**2b**)

32 mg, 84% yield; ^1^H-NMR (600 MHz, CDCl_3_): δ 6.36 (d, *J* = 8.6 Hz, 1H, N***H***CO), 5.20–5.17 (m, 1H, ***H_sn-2_***), 4.77 (d, *J* = 3.5 Hz, 1H, ***H-1***), 4.45 (dd, *J* = 11.7, 4.2 Hz, 1H, ***H_sn-1a_***), 4.08 (m, 1H, ***H-2***), 4.04 (dd, *J* = 11.9, 6.0 Hz, 1H, ***H_sn-1b_***), 3.89 (dd, *J* = 11.8, 2.2 Hz, 1H, ***H-6a***), 3.79–3.76 (m, 1H, ***H-6b***), 3.75 (dd, *J* = 11.0, 4.9 Hz, 1H, ***H_sn-3a_***), 3.70–3.64 (m, 2H, ***H-3***, ***H-5***), 3.56–3.54 (m, 1H, ***H-4***), 3.52 (dd, *J* = 11.0, 5.6 Hz, 1H, ***H_sn-3b_***), 2.31–2.21 (m, 6H, 3 × CO–C***H_2_***), 1.60–1.57 (m, 6H, 3 × CO–CH_2_–C***H_2_***), 1.33–1.23 (m, 52H, CH_2_–(C***H_2_***)_2_–CH_3_, 2 × CH_2_–CH_2_–(C***H_2_***)_12_–CH_3_), 0.92–0.84 (m, 9H, 3 × C***H_3_***); ^13^C-NMR (126 MHz, CDCl_3_): δ 175.40, 173.48, 173.10, 97.91, 73.27, 72.00, 70.50, 69.81, 65.66, 61.94, 61.34, 53.42, 36.35, 34.27, 34.08, 31.92, 31.44, 30.54, 29.70–29.14, 25.26, 24.92, 24.87, 22.68, 22.38, 14.12, 13.98; HR-ESI-MS *m*/*z* calcd. for C_47_H_90_NO_10_ [M + H]^+^ 828.6559, found 828.6565.

##### 1,2-Dipalmitoyl-3-*O*-(*N*-palmitoyl-2′-amino-2′-deoxy-α-d-glucopyranosyl)-*sn*-glycerol (**2c**)

65 mg, 96% yield; ^1^H-NMR (500 MHz, CDCl_3_): δ 6.35 (d, *J* = 8.1 Hz, 1H, N***H***CO), 5.20–5.17 (m, 1H, ***H_sn-2_***), 4.76 (d, *J* = 3.7 Hz, 1H, ***H-1***), 4.53 (dd, *J* = 11.5, 4.7 Hz, 1H, ***H_sn-1a_***), 4.09–4.05 (m, 1H, ***H-2***), 4.02 (dd, *J* = 11.5, 5.6 Hz, 1H, ***H_sn-1b_***), 3.88–3.82 (m, 2H, ***H-6*** × 2), 3.80 (dd, *J* = 11.1, 4.5 Hz, 1H, ***H_sn-3a_***), 3.69 (t, *J* = 9.3 Hz, 1H, ***H-3***), 3.64–3.56 (m, 2H, ***H-5***, ***H-4***), 3.52 (dd, *J* = 11.3, 5.4 Hz, 1H, ***H_sn-3b_***), 2.36–2.27 (m, 6H, 3 × CO–C***H_2_***), 1.61–1.58 (m, 6H, 3 × CO–CH_2_–C***H_2_***), 1.30–1.25 (m, 72H, 3 × CH_2_–CH_2_–(C***H_2_***)_12_–CH_3_), 0.88 (t, *J* = 6.9 Hz, 9H, 3 × C***H_3_***); ^13^C-NMR (126 MHz, CDCl_3_): δ 178.46, 173.54, 173.16, 97.77, 74.06, 71.72, 71.42, 69.83, 65.63, 62.01, 61.72, 53.58, 34.29, 34.09, 33.87, 31.91, 29.70–29.08, 25.62, 24.92, 24.88, 24.72, 22.68, 14.10; HR-MALDI-MS calcd. for C_57_H_109_NO_10_ Na [M + Na]^+^ 990.7949, found 990.7952.

##### 1,2-*O*-Dipalmitoyl-3-*O*-(6′-*O*-palmitoyl-α-d-glucopyranosyl)-*sn*-glycerol (**3a**)

To a solution of **25** (58 mg, 0.11 mmol) in dry pyridine (5 mL), DMAP (3 mg, 0.02 mmol) and palmitoyl chloride (0.3 mL, 0.67 mmol) were added at 80 °C. The reaction mixture was stirred for 4 h, and then was concentrated and diluted with CH_2_Cl_2_ (30 mL) and washed sequentially with 1 M HCl and saturated NaHCO_3_. The organic phase was dried over Na_2_SO_4_, filtrated, concentrated. Purification by flash chromatography (AcOEt-petroleum ether 1:8) gave a crude. To a solution of the crude in 5 mL AcOEt/MeOH (1:1), 20% palladium hydroxide (80 mg) was added and stirred at r.t. under hydrogen atmosphere for 1 h. After filtration the solvent was evaporated and the residue was purified by silica column chromatography (AcOEt-petroleum ether 1:1) to afford **3a** (66 mg, 62% for 2 steps) as a white solid. ^1^H-NMR (600 MHz, CDCl_3_): 5.26–5.23 (m, 1H, ***H_sn-2_***), 4.86 (d, *J* = 3.9 Hz, 1H, ***H-1***), 4.55 (dd, *J* = 12.2, 4.0 Hz, 1H, ***H-6a***), 4.40 (dd, *J* = 11.8, 4.3 Hz, 1H, ***H_sn-1a_***), 4.19 (dd, *J* = 12.3, 2.1 Hz, 1H, ***H-6b***), 4.12 (dd, *J* = 11.9, 5.9 Hz, 1H, ***H_sn-1b_***), 3.84 (dd, *J* = 11.0, 4.6 Hz, 1H, ***H_sn-3a_***), 3.74–3.71 (m, 2H, ***H-3***, ***H-5***), 3.61 (dd, *J* = 11.0, 6.0 Hz, 1H, ***H_sn-3b_***), 3.50 (dd, *J* = 9.3, 3.7 Hz, 1H, ***H-2***), 3.34 (t, *J* = 9.5 Hz, 1H, ***H-4***), 2.37–2.29 (m, 6H, C***H_2_*** × 3), 1.63–1.60 (m, 6H, C***H_2_*** × 3***)***, 1.29–1.24 (m, 72H, (C***H_2_***)_12_ × 3), 0.88 (t, *J* = 7.0 Hz, 9H, C***H_3_*** × 3); HR-MALDI-MS *m*/*z* calcd. for C_57_H_108_O_11_ Na [M + Na]^+^ 991.7784, found 991.7783.

##### General Procedure for Compounds **3b**–**3e**

Pd(OH)_2_/C (20%) was added to a mixture of compounds **30**–**32** in AcOEt/MeOH (1:1). Upon stirring for 4 h under H_2_, the mixture was filtered off and the solvent was evaporated. After purification by column chromatography (CH_2_Cl_2_/MeOH 10:1), compounds **3c**–**3e** were afforded as white solids.

Under the hydrogenation-reduction method mentioned above, only deiodinated product was found from the starting material **29**. After a quick purification by column chromatography (petroleum ether/AcOEt 12:1), the deiodinated product converted to the target compound **3b** as white solid by hydrogenation-reduction once more.

##### 1,2-Dipalmitoyl-3-*O*-(6-deoxy-α-d-glucopyranosyl)-*sn*-glycerol (**3b**)

78 mg, 81% yield; ^1^H-NMR (600 MHz, CDCl_3_): δ5.27–5.22 (m, 1H, ***H_sn-2_***), 4.77 (d, *J* = 3.6 Hz, 1H, ***H-1***), 4.38 (dd, *J* = 12.0, 3.7 Hz, 1H, ***H_sn-1a_***), 4.16 (dd, *J* = 12.0, 6.1 Hz, 1H, ***H_sn-1b_***), 3.80 (dd, *J* = 10.7, 5.4 Hz, 1H, ***H_sn-3a_***), 3.67 (t, *J* = 9.3, 1H, ***H-3***), 3.65–3.60 (m, 1H, ***H-5***), 3.58 (dd, *J* = 10.7, 5.8 Hz, 1H, ***H_sn-3b_***), 3.48 (dd, *J* = 9.1, 3.3 Hz, 1H, ***H-2***), 3.13 (t, *J* = 9.3 Hz, 1H, ***H-4***), 2.32–2.28 (m, 4H, 2 × CO–C***H_2_***), 1.62–1.58 (m, 4H, 2 × CO–CH_2_–C***H_2_***), 1.31–1.24 (m, 51H, 2 × CH_2_–CH_2_–(C***H_2_***)_12_–CH_3_, C***H_3_***), 0.87 (t, *J* = 7.0 Hz, 6H, 2 × C***H_3_***); ^13^C-NMR (150 MHz, CDCl_3_): δ 173.9, 173.4, 98.8, 75.4, 74.1, 72.3, 69.8, 67.7, 65.9, 62.4, 34.4, 34.2, 32.0, 29.6–29.2, 25.0, 24.9, 22.8, 17.7, 14.2; HR-ESI-MS *m*/*z* calcd. for C_41_H_79_O_9_ [M + H]^+^ 715.5719, found 715.5709.

##### 1,2-Dipalmitoyl-3-*O*-α-d-glucosyluronate-*sn*-glycerol (**3c**)

84 mg, 76% yield; ^1^H-NMR (600 MHz, DMSO-D6): δ 5.12–5.10 (m, 1H, ***H_sn-2_***), 4.70 (d, *J* = 3.1 Hz, 1H, ***H-1***), 4.33 (dd, *J* = 11.7, 1.5 Hz, 1H, ***H_sn-1a_***), 4.22 (dd, *J* = 12.1, 6.6 Hz, 1H, ***H_sn-1b_***), 3.81 (d, *J* = 9.9 Hz, 1H, ***H-5***), 3.70 (dd, *J* = 11.0, 4.9 Hz, 1H, ***H_sn-3a_***), 3.55 (dd, *J* = 11.4, 5.4 Hz, 1H, ***H_sn-3b_***), 3.39 (t, *J* = 9.1, 1H, ***H-3***), 3.31 (t, *J* = 9.4 Hz, 1H, ***H-4***), 3.23 (dd, *J* = 9.2, 3.1 Hz, 1H, ***H-2***), 2.30–2.22 (m, 4H, 2 × CO–C***H_2_***), 1.51–1.48 (m, 4H, 2 × CO–CH_2_–C***H_2_***), 1.34–1.20 (m, 48H, 2 × CH_2_–CH_2_–(C***H_2_***)_12_–CH_3_), 0.84 (t, *J* = 6.8 Hz, 6H, 2 × C***H_3_***); ^13^C-NMR (150 MHz, CDCl_3_): δ 172.3, 172.0, 170.7, 99.2, 72.3, 71.7, 71.4, 71.1, 69.3, 65.3, 61.9, 33.3, 33.2, 31.1, 28.9–28.2, 24.2, 24.1, 21.9, 13.6; HR-ESI-MS *m*/*z* calcd. for C_41_H_76_O_11_ Na [M + Na]^+^ 767.5280, found 767.5274.

##### 1,2-Dipalmitoyl-3-*O*-(6′-*O*-sulfonato-α-d-glucopyranoside)-*sn*-glycerol (**3d**)

46 mg, 88% yield; ^1^H-NMR (600 MHz, DMSO-D6): δ 5.14–5.10 (m, 1H, ***H_sn-2_***), 4.63 (d, *J* = 2.8 Hz, 1H, ***H-1***), 4.33–4.28 (m, 1H, ***H_sn-1a_***), 4.14–4.10 (m, 1H, ***H_sn-1b_***), 3.95–3.92 (m, 1H, ***H_sn-3a_***), 3.78–3.75 (m, 1H, ***H_sn-3b_***), 3.69–3.66 (m, 1H, ***H-6a***), 3.48–3.35 (m, 3H, ***H-2***, ***H-5***, ***H-6b***), 3.19–3.16 (m, 1H, ***H-3***), 3.08–3.03 (m, 1H, ***H-4***), 2.29–2.23 (m, 4H, 2 × CO–C***H_2_***), 1.53–1.49 (m, 4H, 2 × –C***H_2_***), 1.26–1.18 (m, 48H, (C***H_2_***)_12_ × 2), 0.85–0.82 (m, 6H, 2 × C***H_3_***); ^13^C-NMR (126 MHz, DMSO-D6): δ 172.88, 172.61, 99.42, 73.20, 72.13, 71.18, 70.45, 69.93, 66.10, 65.53, 62.73, 34.05, 33.89, 31.79, 29.57–28.95, 24.89, 22.58, 14.35; LR-ESI-MS *m*/*z* calcd. for C_41_H_77_O_13_S [M − H]^−^ 809.5, found 809.7.

##### 1,2-Dipalmitoyl-3-*O*-[α-d-galactopyranosyl-(1′′→6′)-α-d-glucopyranosyl]-*sn*-glycerol (**3e**)

109 mg, 89% yield; ^1^H-NMR (500 MHz, CDCl_3_/CD_3_OD = 1:1): δ 5.20–5.18 (m, 1H, ***H_sn-2_***), 4.86 (d, *J* = 2.7 Hz, 1H, ***H-1′′***), 4.78 (d, *J* = 3.4 Hz, 1H, ***H-1′***), 4.37 (dd, *J* = 12.0, 3.1 Hz, 1H, ***H_sn-1a_***), 4.11 (dd, *J* = 12.0, 6.6 Hz, 1H, ***H_sn-1b_***), 4.00 (dd, *J* = 10.9, 2.8 Hz, 1H, ***H_sn-3a_***), 3.94 (ds, 1H, ***H-4′′***), 3.81 (m, 1H, ***H-3′′***), 3.77 (dd, *J* = 10.9, 5.3 Hz, 1H, ***H-6a′***), 3.73–3.69 (m, 3H, ***H-2′′***, ***H-5′′***, ***H-6a′′***), 3.64–3.55 (m, 4H, ***H-3′***, ***H-5′***, ***H-6b′***, ***H_sn-3b_***), 3.53–3.45 (m, 2H, ***H-4′***, ***H-6b′′***), 3.42 (dd, *J* = 9.5, 3.4 Hz, 1H, ***H-2′***), 2.28–2.24 (m, 4H, O=CC***H_2_*** × 2), 1.56–1.52 (m, 4H, O=CCH_2_C***H_2_*** × 2), 1.28–1.20 (m, 48H, (C***H_2_***)_12_ × 2), 0.83 (t, *J* = 6.9 Hz, 6H, C***H_3_*** × 2); ^13^C-NMR (126 MHz, CDCl_3_): δ 173.87, 173.58 (O=C × 2), 99.42 (C-1′), 98.64 (C-1′′), 73.73 (C-5′), 71.81 (C-2′), 70.96 (C-3′), 70.51 (C-3′′), 70.32 (C-4′), 69.97 (C_sn-2_), 69.57 (C-5′′), 69.38 (C-2′′), 69.20 (C-4′′), 66.30 (C_sn-3_), 65.56 (C-6′), 62.50 (C_sn-1_), 61.39 (C-6′′), 34.18, 34.02 (COCH_2_ × 2), 31.83 (COCH_2_CH_2_ × 2), 29.61–29.03 (CH_2_ × 20), 24.82, 24.80 (CH_2_ × 2), 22.58 (CH_2_ × 2), 13.93 (CH_3_ × 2); HR-ESI-MS *m*/*z* calcd. for C_47_H_88_O_15_ Na [M + Na]^+^ 915.6015, found 915.6012.

##### General procedures for **4a**–**4c**

Pd(OH)_2_/C (20%) was added to a mixture of compounds **35a**–**35c** in THF/MeOH (1:1). Upon stirring for 4 h under H_2_, the mixture was filtered off and the solvent was evaporated. After purification by column chromatography (CH_2_Cl_2_/MeOH 20:1), compounds **4a**–**4c** were afforded as white solids.

##### Bis(tetradecyl)methyl-*N*-acetyl-6-amino-6-deoxy-α-d-glucopyranoside (**4a**)

25 mg, 89% yield; ^1^H-NMR (500 MHz, CDCl_3_): δ 5.87 (dd, *J* = 7.5, 4.8 Hz, 1H, -N***H***), 4.89 (d, *J* = 4.2 Hz, 1H, ***H-1***), 4.07–4.02 (m, 1H, ***H-6a***), 3.73 (t, *J* = 9.3 Hz, 1H, ***H-3***), 3.69–3.66 (m, 1H, ***H-5***), 3.58–3.53 (m, 1H, OC***H***), 3.48 (dd, *J* = 9.2, 3.9 Hz, 1H, ***H-2***), 3.13 (t, *J* = 9.4 Hz, 1H, ***H-4***), 3.01–2.99 (m, 1H, ***H-6b***), 2.06 (s, 3H, COC***H_3_***), 1.53–1.48 (m, 4H, OCH-(C***H_2_***)_2_), 1.34–1.25 (m, 48H, (C***H_2_***)_12_ × 2), 0.88 (t, *J* = 6.7 Hz, 6H, C***H_3_*** × 2); ^13^C-NMR (126 MHz, CDCl_3_): δ 172.65 (C=O), 97.49 (C-1), 78.96 (O–C), 73.71 (C-3), 72.69 (C-2), 71.15 (C-5), 70.07 (C-4), 40.01 (C-6), 34.57, 33.44, 32.08 (CH_2_ × 2), 29.97, 29.92, 29.85–29.81, 29.78, 29.77, 29.72 29.52 (CH_2_ × 2), 25.62, 25.22, 23.18 (CH_3_), 22.85 (CH_2_ × 2), 14.27 (CH_3_ × 2); HR-ESI-MS *m*/*z* calcd. for C_37_H_74_NO_6_ [M + H]^+^ 628.5511, found 628.5500.

##### Bis(tetradecyl)methyl-*N*-hexanoyl-6-amino-6-deoxy-α-d-glucopyranoside (**4b**)

34 mg, 94% yield; ^1^H-NMR (500 MHz, CDCl_3_): δ 5.88–5.86 (m, 1H, -N***H***), 4.89 (d, *J* = 4.2 Hz, 1H, ***H-1***), 4.07–4.02 (m, 1H, ***H-6a***), 3.72 (t, *J* = 9.1 Hz, 1H, ***H-3***), 3.69–3.66 (m, 1H, ***H-5***), 3.58–3.53 (m, 1H, OC***H***), 3.47 (dd, *J* = 9.1, 3.7 Hz, 1H, ***H-2***), 3.09 (t, *J* = 9.4 Hz, 1H, ***H-4***), 3.01–2.98 (m, 1H, ***H-6b***), 2.26–2.22 (m, 2H, COC***H_2_***), 1.67–1.62 (m, 2H, COCH_2_C***H_2_***), 1.51–1.46 (m, 4H, OCH-(C***H_2_***)_2_), 1.33–1.25 (m, 52H, (C***H_2_***)_2_, (C***H_2_***)_12_ × 2), 0.96–0.86 (m, 9H, C***H_3_*** × 3). ^13^C-NMR (126 MHz, CDCl_3_): δ 175.80 (C=O), 97.48 (C-1), 78.91 (O–C), 73.66 (C-3), 72.70 (C-2), 71.20 (C-5), 70.13 (C-4), 39.84 (C-6), 36.60, 34.57, 33.43, 32.08 (CH_2_ × 2), 31.57, 29.97, 29.92, 29.85–29.72, 29.51 (CH_2_ × 2), 25.62, 25.48, 25.22, 22.84 (CH_2_ × 2), 22.50, 14.27 (CH_3_ × 2), 14.07 (CH_3_); HR-ESI-MS *m*/*z* calcd. for C_41_H_82_NO_6_ [M + H]^+^ 684.6137, found 684.6130.

##### Bis(tetradecyl)methyl-*N*-palmitoyl-6-amino-6-deoxy-α-d-glucopyranoside (**4c**)

43 mg, 96% yield; ^1^H-NMR (500 MHz, CDCl_3_): δ 5.83 (dd, *J* = 8.3, 4.7 Hz, 1H, -N***H***), 4.89 (d, *J* = 4.2 Hz, 1H, ***H-1***), 4.07 (ddd, *J* = 14.4, 8.6, 2.0 Hz, 1H, ***H-6a***), 3.72 (t, *J* = 9.3 Hz, 1H, ***H-3***), 3.68–3.66 (m, 1H, ***H-5***), 3.59–3.53 (m, 1H –OC***H***–), 3.46 (dd, *J* = 9.5, 3.9 Hz, 1H, ***H-2***), 3.08 (t, *J* = 9.5 Hz, 1H, ***H-4***), 3.37 (dt, *J* = 14.1, 4.4 Hz, 1H, ***H-6b***), 2.25 (dt, *J* = 7.0, 1.3 Hz, 2H, –COC***H_2_***–), 1.65–1.62 (m, 2H, COCH_2_C***H_2_***), 1.52–1.48 (m, 4H, OCH(C***H_2_***)_2_), 1.33–1.22 (m, 72H, COCH_2_CH_2_(C***H_2_***)_12_, (C***H_2_***)_12_ × 2), 0.88 (t, *J* = 6.9 Hz, 9H, C***H_3_*** × 3); ^13^C-NMR (126 MHz, CDCl_3_): δ 175.86 (C=O), 97.48 (C-1), 78.92 (O–C), 73.68 (C-3), 72.73 (C-2), 71.22 (C-5), 70.09 (C-4), 39.85 (C-6), 36.65, 34.58, 33.43, 32.08 (CH_2_ × 3), 29.98, 29.92, 29.85–29.77, 29.72, 29.63, 29.52 (CH_2_ × 2), 29.46, 29.44, 25.82, 25.61, 25.22, 22.85 (CH_2_ × 3), 14.28 (CH_3_ × 3), HR-MALDI-MS *m*/*z* calcd. for C_51_H_101_O_6_N Na [M + Na]^+^ 846.7527, found 846.7520.

### 3.2. Biological Methods

#### 3.2.1. Cell Culture and Virus Infection

Madin-Darby canine kidney (MDCK) cells were obtained from the Cell Bank of the Chinese Academy of Sciences (Shanghai, China) and grown in RPM1640 medium (Hangzhou genome biomedical Ltd., Hangzhou, China) supplemented with 10% FBS, 100 units/mL of penicillin and 100 μg/mL of streptomycin. Influenza virus (A/Puerto Rico/8/34 [H1N1]; PR/8) was propagated in 10-d-old embryonated eggs for 3 d at 36.5 °C.

The virus infection experiments *in vitro* were performed as described previously [36]. In brief, virus propagation solution was diluted in PBS containing 0.2% bovine serum albumin and was added to cells at the indicated multiplicity of infection (MOI). Virus was allowed to adsorb for 60 min at 4 °C. After removing the virus inoculum, cells were maintained in infecting media (RPM1640, 4 μg/mL trypsin, Hangzhou genome biomedical Ltd.) at 37 °C in 5% CO_2_.

#### 3.2.2. Infectivity Antiviral Assays

The antiviral activity was evaluated by the cytopathic effects (CPE) inhibition assay. MDCK cell cultures in 96-well plates were firstly infected with IAV (MOI = 1.0), and then treated with different compounds in triplicate after removal of the virus inoculum. After 48 h incubation, the cells were fixed with 4% formaldehyde for 20 min at room temperature. After removal of the formaldehyde, the cells were stained with 0.1% crystal violet for 30 min. The plates were washed and dried, and the intensity of crystal violet staining for each well was measured at 570 nm. The virus inhibition (%) was calculated by the equation:

Virus inhibition (%) = [(Asample_570_ − Avirus_570_)/(Amock_570_ − Avirus_570_)] × 100 (1)

where, Amock_570_ was the absorbance without virus infection, Asample_570_ was absorbance with virus infection and drug treatment, Avirus_570_ was absorbance with virus infection but without drugs.

#### 3.2.3. *In Vivo* Experiments

Four-week-old female Kunming mice (average weight, 13.0 ± 1.0 g) were housed and studied under protocols approved by the Animal Ethics Committee of Ocean University of China. Briefly, mice were randomly divided into different experimental groups (10 mice/group). The drugs administration started 1 d prior to virus inoculation. Virus-infected control group (virus control) and uninfected control group (normal control) received 1 × PBS as a placebo. On the day of virus inoculation, mice were lightly anaesthetized and each was inoculated intranasally with PR8 virus (4 HAU/mouse) diluted in 40 μL of 1 × PBS. Two hours after inoculation, mice received oral administration of **1d** (5 or 10 mg/kg/d) or oseltamivir (20 mg/kg/d), and the treatments were repeated once daily for the entire experiment. Mice were weighed and killed on Day 4 after inoculation, and lungs were removed and weighed. The lung index was calculated by the following equation using the obtained values:

Lung index = [Lung weight (g)/Mice weight (g)] × 100
(2)

By this index, the severity of lung injury in pneumonia mice was evaluated. Subsequently, lung specimens of animals from each experimental group were homogenized in 1 × PBS (pH 7.4) for determination of viral titers by neuraminidase activity assay.

In the survival experiments, 10 mice per group were intranasally infected with PR/8 virus (Wuhan Institute of Virology, Wuhan, China) (6 HAU/mouse) at Day 0. IAV infected mice received oral administration of compound **1d** (5 or 10 mg/kg/d) or oseltamivir (20 mg/kg/d), and the virus control group and normal control group received PBS as a placebo. The drugs administration started 1 d prior to virus infection and was repeated once daily during the course of the experiment, and survival was assessed in all groups for 14 d after infection.

## 4. Conclusions

In this study, four series of glycoglycerolipids (**1**–**4**), seventeen analogs of **AGGL**, were designed and prepared. The successful total synthesis afforded enough samples for anti-IAV screening, and the results indicated that the acylamino and glycerol groups of the glycolipids were essential for the inhibitory effect on IAV multiplication. Furthermore, the potent derivative **1d** was able to significantly improve survival and decrease pulmonary viral titers in IAV-infected mice, which could provide novel insights into deeper exploration of the unique aminoglycoglycerolipids in drug discovery of pneumonia diseases caused by viruses.

## Figures and Tables

**Table 1:** The inhibitory effect of aminoglycoglycerolipids and analogs on IAV replication *in vitro* ^a^.

Sample	Virus Inhibition (%)	Sample	Virus Inhibition (%)	Sample	Virus Inhibition (%)
1a	27.1	2a	6.1	3a	12.0
1b	46.1	2b	50.1	3b	6.4
1c	29.3	2c	19.0	3c	12.5
1d	46.1	4a	4.0	3d	3.7
1e	9.4	4b	2.9	3e	51.5
1f	10.4	4c	0	AGGL	25.5
-	-	-	-	Ribavirin	50.0

^a^ Performed at a concentration of 50 μM.

**Table 2:** The anti-IAV activities of compound **1d** *in vivo*.

Groups	Dose (mg/kg/d)	Lung Index ^a^ (X ± SD)	Inhibitory Rate (%)
Virus control	-	1.24 ± 0.31 #	0
Normal control	-	0.79 ± 0.10	-
Oseltamivir	20	0.87 ± 0.41 *	29.7
1d	5	1.05 ± 0.31	15.3
1d	10	1.15 ± 0.49	7.3

Values are means ± S.D. (*n* = 10); # *p* < 0.05 *vs.* normal control group; *t*-test, * *p* < 0.05 *vs.* virus control group; *t*-test; ^a^ Lung index = [lung weight (g)/mice weight (g)] × 100.

## Supplementary Materials

The following are available online at www.mdpi.com/1660-3397/14/6/116/s1, The Chemical synthesis of compounds **7**, **9**–**11**, **12a**–**12f**, **13**, **14**, **16**, **18**–**20**, **21a**–**21c**, **24**–**27**, **29**–**34**, **35a**–**35c** and their ^1^H-NMR and MS Data.
