# Supplementary material for: Synthesis and Anti-Influenza A Virus Activity of 6′-amino-6′-deoxy-glucoglycerolipids Analogs
Source: Mar Drugs. 2016 Jun 18;14(6):116. doi: 10.3390/md14060116 (PMC4926075; doi:10.3390/md14060116)
Supplement: Supplementary file 1 [file marinedrugs-14-00116-s001.pdf]

Supplementary Materials: Synthesis and Anti-Influenza A Virus Activity of 6'-amino-6'-deoxy-glucoglycerolipids Analogs

Li Ren, Jun Zhang, Haizhen Ma, Linlin Sun, Xiaoshuang Zhang, Guangli Yu, Huashi Guan, Wei Wang and Chunxia Li

p-methylphenyl 2,3,4-tri-*O*-benzyl-6-azido-6-deoxy-1-thio-β-D-glucopyranoside (**7**)

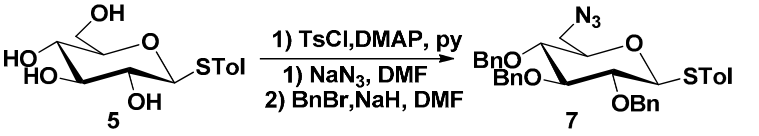

Compound **5** (1.0 g, 3.5 mmol) was dissolved in dry pyridine (10 mL) and tosyl chloride (1.2 g, 6.3 mmol) and DMAP (0.08 g, 0.65 mmol) were added at r.t. After stirring for 4 h the solvent was removed under reduced pressure, then the residue was dissolved in 30 mL CH_2Cl_2 and washed sequentially with 1 M HCl, saturated $NaHCO_3$ solution and brine. The organic layer was dried with Na_2SO_4 and concentrated. The residue was purified by silica gel chromatography (AcOEt-petroleum ether 1:1) to give a white solid (1.2 g, 79%). To the solution of the white solid (3.2 g, 7.5 mmol) in dry DMF (15 mL) was added NaN_3 (2.0 g, 30.0 mmol). The reaction mixture was stirred at 80 °C for 5 h, and then cooled to r.t. The mixture solution was filtered, and then NaH (1.1 g, 25.7 mmol) and BnBr (3.1 mL, 25.7 mm) were added to the filtrate to continue the reaction. After stirring for 1 h, the solution was diluted with brine and extracted with CH_2Cl_2. All of the organic extracts were combined, dried and concentrated under vacuum to give a residue. The residue was purified by silica gel chromatography (AcOEt-petroleum ether 1:10) to give **7** (3.6 g, 86% over two steps) as a white solid. ^{1}H-NMR (600 MHz, $CDCl_3$): δ 7.52 (d, *J* = 8.1 Hz, 2H, STol Ar***H***), 7.40–7.26 (m, 15H, Ar***H***), 7.15 (d, *J* = 8.0 Hz, 2H, STol Ar***H***), 4.97–4.61 (m, 6H, PhC***H***$_2$ × 3), 4.62 (d, *J* = 9.8 Hz, 1H, ***H-1***), 3.72 (t, *J* = 8.9 Hz, 1H, ***H-3***), 3.57–3.52 (m, 2H, ***H-6a***, ***H-4***), 3.49 (t, *J* = 9.2 Hz, 1H, ***H-2***), 3.45 (ddd, *J* = 9.6, 5.5, 2.2 Hz, 1H, ***H-5***), 3.37 (dd, *J* = 13.1, 5.5 Hz, 1H, ***H-6b***), 2.37 (s, 3H, STol ***CH***$_3$); LR-ESI-MS *m*/*z* calcd. for $C_{34}H_{35}N_3O_4S$ Na $[M + Na]^+$ 604.2, found 604.3.

3-*O*-(6'-azido-2',3',4'-tri-*O*-benzyl-6'-deoxy-*α*-D-glucopyranosyl)-1,2-isopropylidene-*sn*-glycerol (**9**)

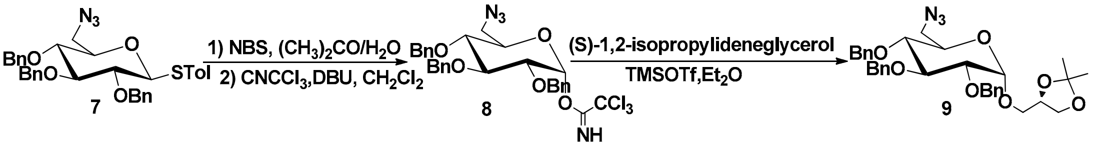

To a stirred solution of **7** (3.8 g, 6.5 mmol) in acetone (27 mL) and H_2O (3 mL) at ambient temperature, NBS (3.4 g, 19.5 mmol) was added. After 0.5 h, the reaction was quenched with saturated $NaHCO_3$ and the mixture was concentrated. The crude intermediate was diluted with CH_2Cl_2 (30 mL) and washed sequentially with saturated $NaHCO_3$, brine, dried over Na_2SO_4 and concentrated. The residue was purified by silica column chromatography to afford oil (3.0 g). To a stirred solution of the oil (1.0 g, 2.1 mmol) in CH_2Cl_2 (8 mL) were added CCl_3CN (840 μL, 8.4 mmol) and DBU (250 μL, 1.6 mmol) at 0 °C. After 0.5 h, the solution was concentrated and the residue was purified by silica column chromatography (AcOEt–petroleum ether 1:5) to afford **8** (1.2 g, 89% for two steps) as a syrup.

A mixture of the glycosyl donor **8** (2.4 g, 3.6 mmol) and activated 4 Å molecular sieves was stirred in dry Et_2O (10 mL) at 0 °C under N_2 atmosphere, then acceptor (*S*)-1,2-isopropylideneglycerol (600 μL, 4.3 mmol) and catalytic amounts of TMSOTf (150 μL, 0.7 mmol) were added. The reaction mixture was stirred at 0 °C under an N_2 atmosphere for 30 min, when TLC showed that the reaction was completed. The mixture was quenched by addition of Et_3N and filtered to remove the sieves. The filtrate was concentrated and the residue was dissolved in 50 mL CH_2Cl_2. The solution was washed with saturated $NaHCO_3$ and brine. The organic layer was dried over Na_2SO_4, then the solvent was

removed *in vacuo*, and the residue was purified by silica column chromatography (AcOEt-petroleum ether 1:7) to afford **9** (2.2 g, 97%, $\alpha/\beta = 12/1$) as an oil. ^{1}H-NMR (600 MHz, $CDCl_3$) α isomer: 7.35–7.31 (m, 15H, Ar***H***), 4.99 (d, *J* = 10.8 Hz, 1H, PhC***H***), 4.90 (d, *J* = 11.0 Hz, 1H, PhC***H***), 4.87 (d, *J* = 3.7 Hz, 1H, ***H-1***), 4.79 (d, *J* = 10.8 Hz, 1H, PhC***H***), 4.77 (d, *J* = 11.9 Hz, 1H, PhC***H***), 4.67 (d, *J* = 11.9 Hz, 1H, PhC***H***), 4.57 (d, *J* = 11.1 Hz, 1H, PhC***H***), 4.39–4.34 (m, 1H, $H_{sn\text{-}2}$), 4.08 (dd, *J* = 8.4, 6.4 Hz, 1H, $H_{sn\text{-}3a}$), 3.96 (t, *J* = 9.3 Hz, 1H, ***H-3***), 3.82 (ddd, *J* = 10.0, 5.6, 2.5 Hz, 1H, ***H-5***), 3.74 (dd, *J* = 8.3, 6.2 Hz, 1H, $H_{sn\text{-}3b}$), 3.64 (dd, *J* = 10.6, 5.6 Hz, 1H, $H_{sn\text{-}1a}$), 3.58–3.52 (m, 2H, ***H-2***, $H_{sn\text{-}1b}$), 3.47–3.42 (m, 2H, ***H-4***, ***H-6a***), 3.33 (dd, *J* = 13.1, 5.5 Hz, 1H, ***H-6b***), 1.43 (s, 3H, C***H***3), 1.37 (s, 3H, C***H***3); LR-ESI-MS calcd. for $C_{33}H_{39}N_3O_7$ Na [M + Na]$^+$ 612.3, found 612.3.

1,2-Dipalmitoyl-3-*O*-(6'-azido-2',3',4'-tri-*O*-benzyl-6'-deoxy-*α*-D-glucopyranosyl)-*sn*-glycerol (**10**)

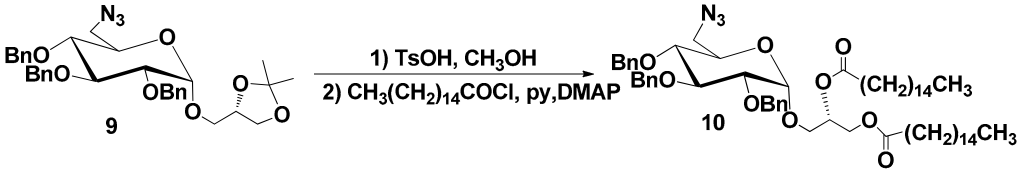

TsOH·H_2O (0.25 g, 0.7 mmol) was added to a stirred solution of **9** (0.9 g, 1.5 mmol) in MeOH (10 mL). After stirring for 2 h at r.t, the solution was concentrated and purified by silica column chromatography (AcOEt-petroleum ether 1:2) to afford the residue (0.8 g). To a solution of the residue (0.3 g, 0.55 mmol) in dry pyridine (25 mL), DMAP (57 mg, 0.3 mmol) and palmitoyl chloride (0.9 mL, 3.0 mmol) were added at 80 °C. The reaction mixture was stirred for 1 h, and then concentrated and diluted with CH_2Cl_2 (30 mL) and washed sequentially with 1 M HCl and saturated $NaHCO_3$. The organic phase was dried over Na_2SO_4, filtrated, concentrated. Purification by flash chromatography (AcOEt-petroleum ether 1:12) yielded compound **10** (0.54 g, 85% for 2 steps) as a white solid. ^{1}H-NMR (600 MHz, $CDCl_3$): δ 7.34–7.31 (m, 15H, Ar***H***), 5.24–5.16 (m, 1H, $H_{sn\text{-}2}$), 4.97(d, *J* = 10.8 Hz, 1H, PhC***H***), 4.89 (d, *J* = 11.0 Hz, 1H, PhC***H***), 4.80 (d, *J* = 10.8 Hz, 1H, PhC***H***), 4.74 (d, *J* = 12.0 Hz, 1H, PhC***H***), 4.73 (d, *J* = 3.6 Hz, 1H, ***H-1***), 4.63 (d, *J* = 11.9 Hz, 1H, PhC***H***), 4.57 (d, *J* = 11.1 Hz, 1H, PhC***H***), 4.41 (dd, *J* = 12.0, 3.7 Hz, 1H, $H_{sn\text{-}3a}$), 4.20 (dd, *J* = 11.9, 6.0 Hz, 1H, $H_{sn\text{-}3b}$), 3.94 (t, *J* = 9.2 Hz, 1H, ***H-3***), 3.78–3.74 (m, 2H, ***H-5***, $H_{sn\text{-}1a}$), 3.56 (dd, *J* = 10.8, 5.5 Hz, 1H, ***H-2***), 3.53 (dd, *J* = 9.5, 3.6 Hz, 1H, $H_{sn\text{-}1b}$), 3.45–3.40 (m, 2H, ***H-4***, ***H-6a***), 3.31 (dd, *J* = 13.2, 5.5 Hz, 1H, ***H-6b***), 2.32–2.27 (m, 4H, 2 × CO–C***H***2), 1.65–1.60 (m, 4H, 2×CO–CH_2–C***H***2), 1.33–1.22 (m, 48H, 2 × CH_2–CH_2–(C***H***2)12–CH_3), 0.88 (t, *J* = 9.2 Hz, 6H, 2 × C***H***3); LR-MALDI-MS *m*/*z* calcd. for $C_{62}H_{95}O_9N_3$ Na [M + Na]$^+$ 1048.7, found 1048.6.

1,2-Dipalmitoyl-3-*O*-(6'-amino-2',3',4'-tri-*O*-benzyl-6'-deoxy-*α*-D-glucopyranosyl)-*sn*-glycerol (**11**)

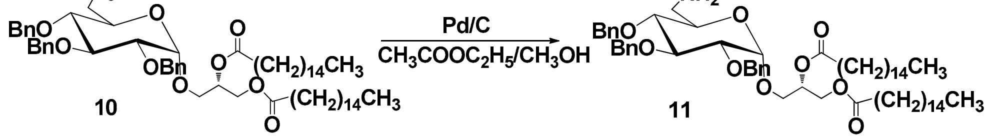

To a solution of **10** (0.20 g, 0.20 mmol) in 20 mL AcOEt/MeOH (1:1) was treated with 10% palladium (0.1 g) and stirred at r.t. under hydrogen atmosphere for 1 h. After filtration the solvent was evaporated and the residue was purified by silica column chromatography (CH_2Cl_2–MeOH 20:1) to afford **11** (0.18 g, 95%) as a paste. ^{1}H-NMR (600 MHz, $CDCl_3$): δ 7.34–7.31 (m, 15H, Ar***H***), 5.28–5.20 (m, 1H, $H_{sn\text{-}2}$), 4.97 (d, *J* = 10.8 Hz, 1H, PhC***H***), 4.87 (d, *J* = 11.1 Hz, 1H, PhC***H***), 4.80 (d, *J* = 10.9 Hz, 1H, PhC***H***), 4.74 (d, *J* = 11.9 Hz, 1H, PhC***H***), 4.69 (d, *J* = 3.5 Hz, 1H, ***H-1***), 4.62 (d, *J* = 11.9 Hz, 1H, PhC***H***), 4.61 (d, *J* = 11.1 Hz, 1H, PhC***H***), 4.41 (dd, *J* = 12.0, 3.6 Hz, 1H, $H_{sn\text{-}3a}$), 4.19 (dd, *J* = 12.0, 6.1 Hz, 1H, $H_{sn\text{-}3b}$), 3.95 (t, *J* = 9.2 Hz, 1H, ***H-3***), 3.73 (dd, *J* = 10.8, 5.7 Hz, 1H, $H_{sn\text{-}1a}$), 3.58–3.54 (m, 1H, ***H-5***), 3.53 (dd, *J* = 10.9, 5.6 Hz, 1H, $H_{sn\text{-}1b}$), 3.48 (dd, *J* = 9.7, 3.7 Hz, 1H, ***H-2***), 3.33 (t, *J* = 9.4 Hz, 1H, ***H-4***), 3.18–3.16 (m, 2H, N***H***2), 2.97 (dd, *J* = 13.4, 2.8 Hz, 1H, ***H-6a***), 2.72 (dd, *J* = 13.5, 6.3 Hz, 1H, ***H-6b***), 2.31–2.26 (m, 4H, 2 × CO–C***H***2), 1.65–1.60 (m, 4H, 2 × CO–CH_2–C***H***2), 1.33–1.22 (m, 48H, 2 × CH_2–CH_2–(C***H***2)12–CH_3), 0.88 (t, *J* = 7.0 Hz, 6H, 2 × C***H***3); LR-ESI-MS *m*/*z* calcd. for $C_{62}H_{97}NO_9$ [M + H]$^+$ 1001.4, found 1001.4.

General procedure for compounds **12a–12f**

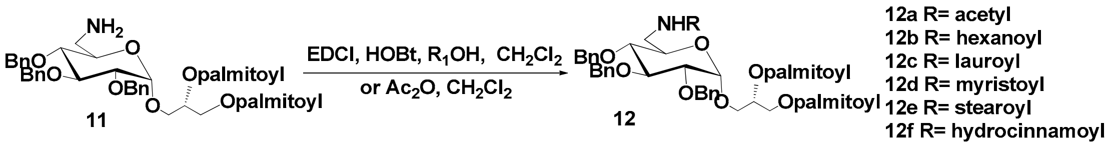

To a solution of compound **11** (100 mg, 0.1 mmol) in CH_2Cl_2 (10 mL) was added Ac_2O (9.5 μL, 0.1 eq) at 0 °C. After stirring for 0.5 h, the mixture was washed with aq. $NaHCO_3$ and brine. The organic layer was dried over Na_2SO_4 and concentrated. The residue was chromatographed (AcOEt–petroleum ether 1:3) to afford **12a** (92 mg, 89%) as a white solid.

To a solution of compound **11** (200 mg, 0.2 mmol) in CH_2Cl_2 (10 mL) were sequentially added EDCI (42 mg, 0.22 mmol), HOBt (30 mg, 0.22 mmol) and corresponding fatty acids (hexadecanoic acid, lauric acid, myristic acid acid, palmitic acid, stearic acid, hydrocinnanmic acid, 1.1 eq) at 0 °C. After stirring for 1 h, the mixture was diluted with aq. $NaHCO_3$ and brine. The organic layer was dried over Na_2SO_4 and concentrated. The residue was chromatographed by silica gel (AcOEt–petroleum ether 1:4) to afford **12b–12f** (41%–89%) as white solids.

1,2-Dipalmitoyl-3-*O*-(*N*-acetyl-6'-amino-2',3',4'-tri-*O*-benzyl-6'-deoxy-*α*-D-glucopyranosyl)-*sn*-glycerol (**12a**)

92 mg, 89%; ^{1}H-NMR (600 MHz, $CDCl_3$): δ 7.37–7.27 (m, 15H, Ar***H***), 5.69 (dd, *J* = 7.2, 4.0 Hz, 1H, N***H***–CO), 5.25–5.20 (m, 1H, $\boldsymbol{H_{sn\text{-}2}}$), 4.97 (d, *J* = 10.8 Hz, 1H, PhC***H***), 4.85 (d, *J* = 10.5 Hz, 1H, PhC***H***), 4.82 (d, *J* = 10.8 Hz, 1H, PhC***H***),4.76 (d, *J* = 12.0 Hz, 1H, PhC***H***), 4.68 (d, *J* = 3.5 Hz, 1H, ***H-1)***, 4.64–4.61 (m, 2H, 2 × PhC***H***), 4.39 (dd, *J* = 12.0, 3.6 Hz, 1H, $\boldsymbol{H_{sn\text{-}1a}}$), 4.18 (dd, *J* = 12.0, 6.2 Hz, 1H, $\boldsymbol{H_{sn\text{-}1b}}$), 3.95 (t, *J* = 9.3 Hz, 1H, ***H-3***) 3.74–3.63 (m, 3H, ***H-5***, $\boldsymbol{H_{sn\text{-}3a}}$, ***H-6a***), 3.59 (dd, *J* = 11.0, 5.6 Hz, 1H, $\boldsymbol{H_{sn\text{-}3b}}$), 3.47 (dd, *J* = 9.7, 3.5 Hz, 1H, ***H-2***), 3.33 (dt, *J* = 13.8, 3.7 Hz, 1H, ***H-6b***), 3.27 (t, *J* = 9.4 Hz, 1H, ***H-4***), 2.31–2.28 (m, 4H, 2 × CO–C$\boldsymbol{H_2}$), 1.92 (s, 1H, NHCOC$\boldsymbol{H_3}$), 1.63–1.55 (m, 4H, 2 × CO–CH_2–C$\boldsymbol{H_2}$), 1.33–1.22 (m, 48H, 2 × CH_2–CH_2–(C$\boldsymbol{H_2}$)$_{12}$–CH_3), 0.88 (t, *J* = 7.0 Hz, 6H, 2 × C$\boldsymbol{H_3}$); LR-ESI-MS *m*/*z* calcd. for $C_{64}H_{100}NO_{10}$ $[M + H]^+$ 1042.7, found 1042.8.

1,2-Dipalmitoyl-3-*O*-(*N*-hexanoyl-6'-amino-2',3',4'-tri-*O*-benzyl-6'-deoxy-*α*-D-glucopyranosyl)-*sn*-glycerol (**12b**)

88 mg, 41%; ^{1}H-NMR (600 MHz, $CDCl_3$): δ 7.38–7.27 (m, 15H, Ar***H***), 5.64 (dd, *J* = 7.4, 3.8 Hz, 1H, N***H***–CO), 5.25–5.21 (m, 1H, $\boldsymbol{H_{sn\text{-}2}}$), 4.96 (d, *J* = 10.8 Hz, 1H, PhC***H***), 4.83 (t, *J* = 10.8 Hz, 2H, 2 × PhC***H***), 4.76 (d, *J* = 12.0 Hz, 1H, PhC***H***), 4.69 (d, *J* = 3.6 Hz, 1H, ***H-1)***, 4.62 (d, *J* = 11.5 Hz, 2H, 2 × PhC***H***), 4.40 (dd, *J* = 12.0, 3.6 Hz, 1H, $\boldsymbol{H_{sn\text{-}1a}}$), 4.18 (dd, *J* = 12.0, 6.1 Hz, 1H, $\boldsymbol{H_{sn\text{-}1b}}$), 3.95 (t, *J* = 9.3 Hz, 1H, ***H-3***), 3.79–3.73 (m, 1H, ***H-5***), 3.71–3.65 (m, 2H, $\boldsymbol{H_{sn\text{-}3a}}$, ***H-6a***), 3.57 (dd, *J* = 10.9, 5.4 Hz, 1H, $\boldsymbol{H_{sn\text{-}3b}}$), 3.46 (dd, *J* = 9.7, 3.5 Hz, 1H, ***H-2***), 3.34 (dt, *J* = 13.9, 3.7 Hz, 1H, ***H-6b***), 3.27 (t, *J* = 9.4 Hz, 1H, ***H-4***), 2.32–2.27 (m, 4H, 2 × CO–C$\boldsymbol{H_2}$), 2.12–2.09 (m, 2H, NH–CO–C$\boldsymbol{H_2}$), 1.63–1.55 (m, 6H, 3 × CO–CH_2–C$\boldsymbol{H_2}$), 1.33–1.22 (m, 52H, 2 × CH_2–CH_2–(C$\boldsymbol{H_2}$)$_{12}$–CH_3, CH_2–CH_2–(C$\boldsymbol{H_2}$)$_2$–CH_3), 0.89–0.86 (m, 9H, 3 × C$\boldsymbol{H_3}$); HR-MALDI-MS *m*/*z* calcd. for $C_{68}H_{107}NO_{10}$ Na $[M + Na]^+$ 1120.7793, found 1120.7760.

1,2-Dipalmitoyl-3-*O*-(*N*-lauroyl-6'-amino-2',3',4'-tri-*O*-benzyl-6'-deoxy-*α*-D- glucopyranosyl)-*sn*-glycerol (**12c**)

200 mg, 87%; ^{1}H-NMR (600 MHz, $CDCl_3$): δ 7.38–7.27 (m, 15H, Ar***H***), 5.65 (dd, *J* = 7.4, 3.9 Hz, 1H, N***H***–CO), 5.23–5.21 (m, 1H, $\boldsymbol{H_{sn\text{-}2}}$), 4.96 (d, *J* = 10.7 Hz, 1H, PhC***H***), 4.83 (t, *J* = 10.5 Hz, 2H, 2 × PhC***H***), 4.76 (d, *J* = 12.0 Hz, 1H, PhC***H***), 4.69 (d, *J* = 3.5 Hz, 1H, ***H-1***), 4.62 (d, *J* = 11.5 Hz, 2H, 2 × PhC***H***), 4.40 (dd, *J* = 12.0, 3.6 Hz, 1H, $\boldsymbol{H_{sn\text{-}1a}}$), 4.18 (dd, *J* = 12.0, 6.1 Hz, 1H, $\boldsymbol{H_{sn\text{-}1b}}$), 3.95 (t, *J* = 9.2 Hz, 1H, ***H-3***), 3.79–3.74 (m, 1H, ***H-5***), 3.71–3.65 (m, 2H, $\boldsymbol{H_{sn\text{-}3a}}$, ***H-6a***), 3.57 (dd, *J* = 10.9, 5.5 Hz, 1H, $\boldsymbol{H_{sn\text{-}3b}}$), 3.47 (dd, *J* = 9.7, 3.5 Hz, 1H, ***H-2***), 3.33 (dt, *J* = 13.7, 3.7 Hz, 1H, ***H-6b***), 3.27 (t, *J* = 9.4 Hz, 1H, ***H-4***), 2.32–2.27 (m, 4H, 2 × CO–C$\boldsymbol{H_2}$), 2.12–2.09 (m, 2H, NH–CO–C$\boldsymbol{H_2}$), 1.63–1.55 (m, 6H, 3 × CO–CH_2–C$\boldsymbol{H_2}$), 1.33–1.22 (m, 64H, 2 × CH_2–CH_2–(C$\boldsymbol{H_2}$)$_{12}$–CH_3, CH_2–CH_2–(C$\boldsymbol{H_2}$)$_8$–CH_3), 0.88 (t, *J* = 7.0 Hz, 9H, 3 × C$\boldsymbol{H_3}$) ; HR-MALDI-MS *m*/*z* calcd. for $C_{74}H_{119}NO_{10}$ Na $[M + Na]^+$ 1204.8732, found 1204.8758.

1,2-Dipalmitoyl-3-*O*-(*N*-myristoyl-6'-amino-2',3',4'-tri-*O*-benzyl-6'-deoxy-*α*-D-glucopyranosyl)-*sn*-glycerol (**12d**)

200 mg, 82%; ^{1}H-NMR (600 MHz, $CDCl_3$): δ 7.33–7.27 (m, 15H, Ar***H***), 5.65 (dd, *J* = 7.4, 3.9 Hz, 1H, N***H***–CO), 5.23–5.20 (m, 1H, $\boldsymbol{H_{sn\text{-}2}}$), 4.96 (d, *J* = 10.7 Hz, 1H, PhC***H***), 4.83 (t, *J* = 10.5 Hz, 2H, 2 × PhC***H***), 4.76 (d, *J* = 12.0 Hz, 1H, PhC***H***), 4.69 (d, *J* = 3.5 Hz, 1H, ***H-1)***, 4.63 (d, *J* = 11.4 Hz, 2H, 2 × PhC***H***), 4.40 (dd, *J* = 12.0, 3.6 Hz, 1H, $\boldsymbol{H_{sn\text{-}1a}}$), 4.18 (dd, *J* = 12.0, 6.1 Hz, 1H, $\boldsymbol{H_{sn\text{-}1b}}$), 3.95 (t, *J* = 9.3 Hz, 1H, ***H-3***), 3.79–3.74 (m, 1H, ***H-5***), 3.71–3.65 (m, 2H, $\boldsymbol{H_{sn\text{-}3a}}$, ***H-6a***), 3.57 (dd, *J* = 10.9, 5.4 Hz, 1H, $\boldsymbol{H_{sn\text{-}3b}}$), 3.47 (dd, *J* = 9.6, 3.5 Hz, 1H, ***H-2***), 3.34 (dt, *J* = 13.8, 3.7 Hz, 1H, ***H-6b***), 3.27 (t, *J* = 9.3 Hz, 1H, ***H-4***), 2.32–2.27 (m, 4H, 2 × CO–C$\boldsymbol{H_2}$), 2.12–2.09 (m, 2H, NH–CO–C$\boldsymbol{H_2}$), 1.63–1.55 (m, 6H, 3 × CO–CH_2–C$\boldsymbol{H_2}$), 1.33–1.22 (m, 68H, 2 × CH_2–CH_2–(C$\boldsymbol{H_2}$)$_{12}$–CH_3, CH_2–CH_2–(C$\boldsymbol{H_2}$)$_{10}$–CH_3), 0.88 (t, *J* = 7.0 Hz, 9H, 3 × –C$\boldsymbol{H_3}$); HR-MALDI-MS *m*/*z* calcd. for $C_{76}H_{123}NO_{10}$ Na 1232.9045 $[M + Na]^+$, found 1232.8992.

1,2-Dipalmitoyl-3-*O*-(*N*-stearoyl-6'-amino-2',3',4'-tri-*O*-benzyl-6'-deoxy-*α*-D-glucopyranosyl)-*sn*-glycerol (**12e**)

223 mg, 89%; ^{1}H-NMR (600 MHz, $CDCl_3$): δ 7.38–7.27 (m, 15H, Ar***H***), 5.64 (dd, *J* = 7.5, 3.9 Hz, 1H, N***H***–CO), 5.23–5.19 (m, 1H, $\boldsymbol{H_{sn\text{-}2}}$), 4.96 (d, *J* = 10.8 Hz, 1H, PhC***H***), 4.82 (t, *J* = 10.5 Hz, 2H, 2 × PhC***H***), 4.75 (d, *J* = 12.0 Hz, 1H, PhC***H***), 4.69 (d, *J* = 3.6 Hz, 1H, ***H-1)***, 4.64–4.60 (m, 2H, 2 × PhC***H***), 4.39 (dd, *J* = 12.0, 3.6 Hz, 1H, $\boldsymbol{H_{sn\text{-}1a}}$), 4.17 (dd, *J* = 12.0, 6.2 Hz, 1H, $\boldsymbol{H_{sn\text{-}1b}}$), 3.94 (t, *J* = 9.2 Hz, 1H, ***H-3***), 3.79–3.74 (m, 1H, ***H-5***), 3.69–3.64 (m, 2H, $\boldsymbol{H_{sn\text{-}3a}}$, ***H-6a***), 3.57 (dd, *J* = 10.9, 5.4 Hz, 1H, $\boldsymbol{H_{sn\text{-}3b}}$), 3.46 (dd, *J* = 9.6, 3.5 Hz, 1H, ***H-2***), 3.33 (dt, *J* = 13.8, 3.7 Hz, 1H, ***H-6b***), 3.27 (t, *J* = 9.3 Hz, 1H, ***H-4***), 2.32–2.27 (m, 4H, 2 × CO–C$\boldsymbol{H_2}$), 2.12–2.09 (m, 2H, NH–CO–C$\boldsymbol{H_2}$), 1.63–1.55 (m, 6H, 3 × CO–CH_2–C$\boldsymbol{H_2}$), 1.33–1.22 (m, 76H, 2 × CH_2–CH_2–(C$\boldsymbol{H_2}$)$_{12}$–CH_3, CH_2–CH_2–(C$\boldsymbol{H_2}$)$_{14}$–CH_3), 0.88 (t, *J* = 7.0 Hz, 9H, 3 × C$\boldsymbol{H_3}$); HR-MALDI-MS *m*/*z* calcd. for $C_{80}H_{131}NO_{10}$ Na $[M + Na]^+$ 1288.9665, found 1288.9682.

1,2-Dipalmitoyl-3-*O*-(*N*-hydrocinnamoyl-6'-amino-2',3',4'-tri-*O*-benzyl-6'-deoxy-*α*-D-glucopyranosyl)-*sn*-glycerol (**12f**)

92 mg, 53%; ^{1}H-NMR (600 MHz, $CDCl_3$): δ 7.38–7.27 (m, 15H, Ar***H***), 7.25–7.23 (m, 2H, Ar***H***), 7.18–7.15(m, 3H, Ar***H***), 5.61–5.59 (dd, *J* = 7.6, 3.7 Hz, 1H, N***H***–CO), 5.21–5.17 (m, 1H, $\boldsymbol{H_{sn\text{-}2}}$), 4.96 (d, *J* = 10.8 Hz, 1H, PhC***H***), 4.81 (d, *J* = 10.8 Hz, 1H, PhC***H***), 4.78 (d, *J* = 10.4 Hz, 1H, PhC***H***), 4.75 (d, *J* = 12.0 Hz, 1H, PhC***H***), 4.65 (d, *J* = 3.5 Hz, 1H, ***H-1)***, 4.62 (d, *J* = 12.0 Hz, 1H, PhC***H***), 4.56 (d, *J* = 10.4 Hz, 1H, PhC***H***), 4.37 (dd, *J* = 12.0, 3.6 Hz, 1H, $\boldsymbol{H_{sn\text{-}1a}}$), 4.15 (dd, *J* = 12.0, 6.2 Hz, 1H, $\boldsymbol{H_{sn\text{-}1b}}$), 3.91 (t, *J* = 9.3 Hz, 1H, ***H-3***), 3.79–3.73 (m, 1H, ***H-5***), 3.66–3.59 (m, 2H, $\boldsymbol{H_{sn\text{-}3a}}$, ***H-6a***), 3.54 (dd, *J* = 11.0, 5.5 Hz, 1H, $\boldsymbol{H_{sn\text{-}3b}}$), 3.39 (dd, *J* = 9.6, 3.6 Hz, 1H, ***H-2***), 3.27 (dt, *J* = 13.9, 3.7 Hz, 1H, ***H-6b***), 3.14 (t, *J* = 9.2 Hz, 1H, ***H-4***), 2.98–2.89 (m, 2H, NHCO–C$\boldsymbol{H_2}$), 2.45–2.41 (m, 2H, C$\boldsymbol{H_2}$Ph), 2.29–2.25 (m, 4H, 2 × CO–C$\boldsymbol{H_2}$), 1.60–1.55 (m, 4H, 2 × CO–CH_2–C$\boldsymbol{H_2}$), 1.33–1.22 (m, 48H, 2 × CH_2–CH_2–(C$\boldsymbol{H_2}$)$_{12}$–CH_3), 0.88 (t, *J* = 7.0 Hz, 6H, 2 × –C$\boldsymbol{H_3}$); HR-MALDI-MS *m*/*z* calcd. for $C_{71}H_{105}NO_{10}$ Na 1154.7631 $[M + Na]^+$, found 1154.7638.

1,3,4,6-tetra-*O*-acetyl-2-Azido-2-deoxy-D-glucopyranoside (**13**) [1]

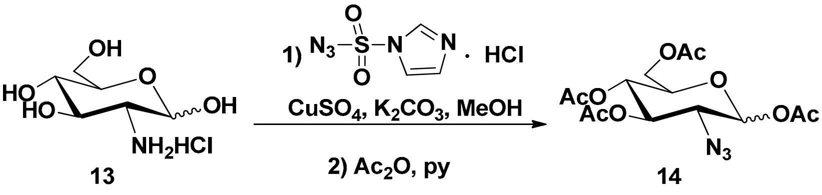

Imidazole-1-sulfonyl Azide Hydrochloride [2] (0.25 g, 1.2 mmol) was added to the mixture of *D*-Glucosamine **13** (6.6 g, 30.0 mmol), K_2CO_3 (9.3 g, 66.5 mmol) and $CuSO_4$. $5H_2O$ (75 mg, 0.3 mmol) in MeOH (120 mL) at r.t. The mixture was stirred for 5 h and concentrated and co-evaporated with toluene. Then Ac_2O (22.0 mL, 240.0 mmol) and DMAP (0.4 g, 3.0 mmol) were added to the residue in pyridine (120 mL) and the mixture was stirred for 4 h. The mixture was concentrated, diluted with H_2O (20 mL) and extracted with AcOEt. The combined organic layer was dried (Na_2SO_4), filtered and concentrated. Flash chromatography (petroleum ether/AcOEt 7:1) gave the **14** (9.2 g, 81%) as a white

solid. ^{1}H-NMR (600 MHz, $CDCl_3$, $\alpha/\beta = 1/6$) β isomer: δ 5.54 (d, *J* = 8.6 Hz, 1H, ***H-1***), 5.08 (t, *J* = 9.6 Hz, 1H, ***H-3***), 5.03 (t, *J* = 9.6, 1H, ***H-4***), 4.29 (dd, *J* = 12.6, 4.5 Hz, 1H, ***H-6a***), 4.07 (dd, *J* = 12.6, 2.1 Hz, 1H, ***H-6b***), 3.8 (ddd, *J* = 9.8, 4.5, 2.1 Hz, 1H, ***H-5***), 3.66 (dd, *J* = 9.9, 8.6 Hz, 1H, ***H-2***), 2.18, 2.08, 2.06, 2.01 (4s, 12H, C***H***$_3$ × 4); LR-ESI-MS *m/z* calcd. for $C_{14}H_{19}N_3O_9Na$ [M + Na]$^+$ 396.1, found 396.1.

p-methylphenyl 3,4,6-tri-*O*-benzyl-2-azido-2-deoxy-1-thio-D-glucopyranoside (**16**)

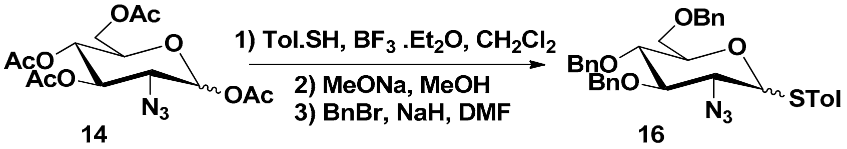

To a solution of peracetylated glucosamine **14** (11.8 g, 31.6 mmol) and *p*-CH_3PhSH (5.8 g, 47.4 mmol) in dry 100 mL CH_2Cl_2, $BF_3{\cdot}Et_2O$ (7.8 mL, 63.2 mmol) was added at 0 °C. The reaction mixture was stirred for 8 h at room temperature and then washed with saturated $NaHCO_3$ and NaCl. The organic layer was dried with Na_2SO_4 and concentrated. The residue was purified by silica column chromatography (petroleum ether-AcOEt, 1:1) to afford colorless oil (9.9 g, 85%). To a solution of the oil (4.8 g, 11.0 mmol) in CH_3OH (30 mL) was added a catalytic amount of NaOMe until pH 9.0. The reaction mixture was stirred for 20 min, and then was neutralized with Amberlite IR120 resin (H^+). After filtration, the filtrate was concentrated *in vacuo* and the residue was dissolved in DMF (40 mL), then NaH (1.7 g, 43.6 mmol) and BnBr (5.2 mL, 43.6 mmol) were added at 0 °C. After stirring for 1 h at room temperature, the mixture was quenched by CH_3OH (10 mL) and concentrated. The residue was dissolved in CH_2Cl_2 (50 mL) and washed with brine, dried over Na_2SO_4 and concentrated. The residue was chromatographed (petroleum ether-AcOEt, 15:1) to give **16** (6.2 g, 98% for 2 steps) as an oil. ^{1}H-NMR (600 MHz, $CDCl_3$, $\alpha/\beta = 3/1$) α isomer: δ 7.52–7.06 (m, 19H, Ar***H***), 5.55 (d, *J* = 5.4 Hz, 1H, ***H-1***), 4.93 (d, *J* = 10.6 Hz, 1H, PhC***H***), 4.89 (d, *J* = 10.5 Hz, 1H, PhC***H***), 4.83 (d, *J* = 10.9 Hz, 1H, PhC***H***), 4.61 (d, *J* = 11.9 Hz, 1H, PhC***H***), 4.55 (d, *J* = 10.8 Hz, 1H, PhC***H***), 4.45 (d, *J* = 11.9 Hz, 1H, PhC***H***), 3.8 (ddd, *J* = 9.9, 3.6, 2.0 Hz, 1H, ***H-5***), 3.95 (dd, *J* = 10.2, 5.4 Hz, 1H, ***H-2***), 3.84 (t, *J* = 10.0 Hz, 1H, ***H-4***), 3.81 (dd, *J* = 10.8, 3.8 Hz, 1H, ***H-6a***), 3.76 (t, *J* = 9.1 Hz, 1H, ***H-3***), 3.66 (dd, *J* = 10.8, 2.0 Hz, 1H, ***H-6b***), 2.33 (s, 3H, STol-C***H***$_3$); LR-ESI-MS *m/z* calcd. for $C_{34}H_{35}N_3O_4S$ Na [M + Na]$^+$ 604.2, found 604.1.

3-*O*-(2'-azide-3',4',6'-tri-*O*-benzyl-2'-deoxy-D-glucopyranosyl)-1,2-isopropylidene-*sn*-glycerol (**18**)

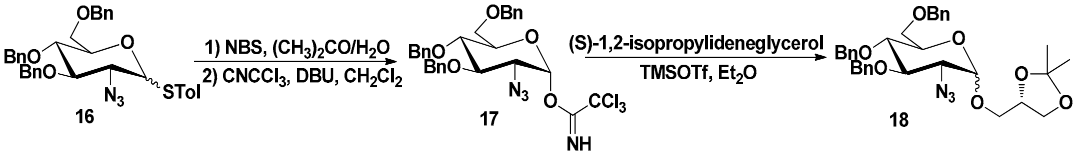

Compound **16** (12.9 g, 22.2 mmol) was treated according to above procedure (synthesis of compound **9**) to give **18** as a yellow oil (9.9 g, 76% for 3 steps). ^{1}H-NMR (500 MHz, $CDCl_3$, $\alpha/\beta = 2:1$):α isomer: δ 7.37–7.15 (m, 15H, Ar***H***), 4.99 (d, *J* = 3.7 Hz, 1H, ***H-1***), 4.89–4.83 (m, 2H, PhC***H*** × 2), 4.81 (d, *J* = 11.0 Hz, 1H, PhC***H***), 4.62 (d, *J* = 12.0 Hz, 1H, PhC***H***), 4.53–4.48 (m, 2H, PhC***H*** × 2), 4.35–4.29 (m, 1H, ***H***$_{sn\text{-}2}$), 4.08 (dd, *J* = 8.2, 6.5 Hz, 1H, ***H***$_{sn\text{-}1a}$), 3.97 (t, *J* = 9.8 Hz, 1H, ***H-3***), 3.85 (d, *J* = 9.8 Hz, 1H, ***H-5***), 3.78 (dd, *J* = 8.3, 6.1 Hz, 1H, ***H***$_{sn\text{-}1b}$), 3.77–3.67 (m, 2H, ***H-4***, ***H-6a***), 3.68 (dd, *J* = 10.3, 5.4 Hz, 1H, ***H-6b***), 3.66 (dd, *J* = 10.6, 1.7 Hz, 1H, ***H***$_{sn\text{-}1a}$), 3.61 (dd, *J* = 10.6, 5.3 Hz, 1H, ***H***$_{sn\text{-}1b}$), 3.37 (dd, *J* = 10.3, 3.4 Hz, 1H, ***H-2***), 1.42 (s, 3H, C***H***$_3$), 1.36 (s, 3H, C***H***$_3$); LR-ESI-MS *m/z* calcd. for $C_{33}H_{39}N_3O_7$ Na [M + Na]$^+$ 612.3, found 612.2.

1,2-Dipalmitoyl-3-*O*-(2'-azide-3',4',6'-tri-*O*-benzyl-2'-deoxy-D-glucopyranosyl)-*sn*-glycerol (**19**)

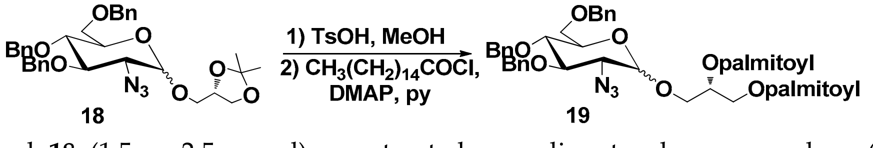

Compound **18** (1.5 g, 2.5 mmol) was treated according to above procedure (synthesis of compound **10**) to give **19** as a yelow oil (2.1 g, 82% for 2 steps). ^{1}H-NMR (600 MHz, $CDCl_3$, $\alpha/\beta = 11/4$)

α isomer: δ 7.37–7.14 (m, 15H, Ar***H***), 5.26–5.22 (m, 1H, $\boldsymbol{H_{sn-2}}$), 4.92 (d, *J* = 3.4 Hz, 1H, ***H-1***), 4.88–4.85 (m, 2H, PhC***H*** × 2), 4.80 (d, *J* = 10.9 Hz, 1H, PhC***H***),4.61 (d, *J* = 12.0 Hz, 1H, PhC***H***), 4.51 (d, *J* = 10.9 Hz, 1H, PhC***H***), 4.48 (d, *J* = 12.0 Hz, 1H, PhC***H***), 4.37 (dd, *J* = 12.0, 3.9 Hz, 1H, $\boldsymbol{H_{sn-1a}}$), 4.16 (dd, *J* = 12.0, 5.9 Hz, 1H, $\boldsymbol{H_{sn-1b}}$), 3.96 (dd, *J* = 10.1, 8.7 Hz, 1H, ***H-3***), 3.83 (dd, *J* = 10.9, 4.8 Hz, 1H, $\boldsymbol{H_{sn-3a}}$), 3.79–3.69 (m, 2H, ***H-5, H-6a***), 3.68–3.62 (m, 3H, ***H-4, H-6b,*** $\boldsymbol{H_{sn-3b}}$), 3.33 (dd, *J* = 10.3, 3.7 Hz, 1H, ***H-2***), 2.35–2.27 (m, 4H, C$\boldsymbol{H_2}$ × 2), 1.63–1.55 (m, 4H, C$\boldsymbol{H_2}$ × 2), 1.33–1.24 (m, 48H, (C$\boldsymbol{H_2}$)$_{12}$ × 2), 0.89 (t, *J* = 6.9 Hz, 6H, C$\boldsymbol{H_3}$ × 2); HR-MALDI-MS *m*/*z* calcd. for $C_{62}H_{95}O_9N_3$ Na [M + Na]$^+$ 1048.6966, found 1048.6967.

1,2-Dipalmitoyl-3-*O*-(2'-amino-3',4',6'-tri-*O*-benzyl-2'-deoxy-*α*-D-glucopyranosyl)-*sn*-glycerol (**20**)

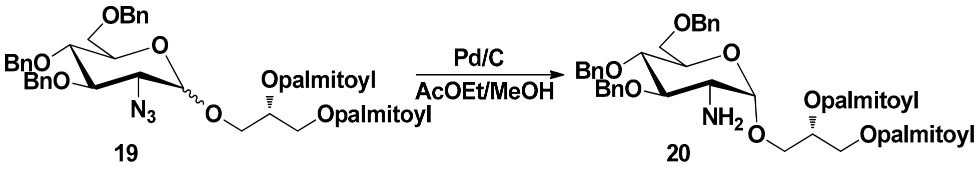

To a solution of **19** (0.5 g, 0.49 mmol) in 12 mL AcOEt/MeOH (1:3) was treated with 10% Pd/C (0.3 g) and stirred at r.t. under hydrogen atmosphere for 2 h. After filtration the solvent was evaporated and the residue was purified by silica column chromatography (CH_2Cl_2–MeOH 20:1) to afford *α*-anomer **20** (0.26 g, 54%) as a white solid. ^{1}H-NMR (600 MHz, $CDCl_3$): δ 7.37–7.16 (m, 15H, Ar***H***), 5.29–5.22 (m, 1H, $\boldsymbol{H_{sn-2}}$), 4.97 (d, *J* = 11.3 Hz, 1H, PhC***H***), 4.85 (d, *J* = 3.4 Hz, 1H, ***H-1***), 4.79 (d, *J* = 10.8 Hz, 1H, PhC***H***), 4.72 (d, *J* = 11.3 Hz, 1H, PhC***H***), 4.65 (d, *J* = 12.1 Hz,1H, PhC***H***), 4.54–4.50 (m, 2H, PhC***H***), 4.34 (dd, *J* = 11.9, 3.9 Hz, 1H, $\boldsymbol{H_{sn-1a}}$), 4.13 (dd, *J* = 11.9, 6.1 Hz, 1H, $\boldsymbol{H_{sn-1b}}$), 3.82 (dd, *J* = 10.8, 4.6 Hz, 1H, $\boldsymbol{H_{sn-3a}}$), 3.78–3.74 (m, 2H, ***H-5, H-6a***), 3.66–3.63 (m, 2H, ***H-3, H-6b***), 3.59 (dd, *J* = 10.8, 6.1 Hz, 1H, $\boldsymbol{H_{sn-3b}}$), 3.54 (t, *J* = 9.5 Hz, 1H, ***H-4***), 2.81 (dd, *J* = 9.9,3.6 Hz, 1H, ***H-2***), 2.32–2.28 (m, 4H, C$\boldsymbol{H_2}$ × 2), 1.63–1.58 (m, 4H, C$\boldsymbol{H_2}$ × 2), 1.33–1.26 (m, 48H, (C$\boldsymbol{H_2}$)$_{12}$ × 2), 0.89 (t, *J* = 9.5 Hz, 6H, C$\boldsymbol{H_3}$ × 2); LR-ESI-MS calcd. for $C_{62}H_{98}NO_9$ [M + H]$^+$ 1000.7, found 1000.7.

General procedure for Compounds **21a–21c**

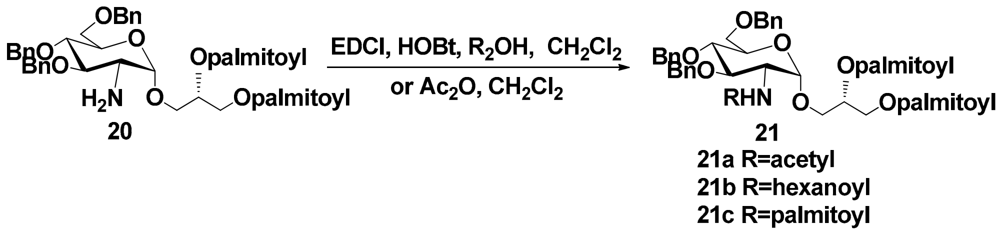

To a solution of compound **20** (100 mg, 0.1 mmol) in CH_2Cl_2 (10 mL) was added Ac_2O (9.5 μL, 0.1 eq) at 0 °C. After stirring for 0.5 h, the mixture was washed with aq. $NaHCO_3$ and brine. The organic layer was dried over Na_2SO_4 and concentrated. The residue was chromatographed (AcOEt-petroleum ether 1:3) to afford **21a** (91 mg, 87%) as a white solid.

To a solution of compound **20** (100 mg, 0.10 mmol) in CH_2Cl_2 (6 mL) were sequentially added EDCI (21 mg, 0.11 mmol), HOBt (15 mg, 0.11 mmol) and corresponding fatty acids (hexadecanoic acid, or palmitic acid, 1.1 eq) at 0 °C. After stirring for 3 h, the mixture was diluted with aq. $NaHCO_3$ and brine. The organic layer was dried over Na_2SO_4 and concentrated. The residue was chromatographed (AcOEt-petroleum ether 1:4–1:6) to afford **21b** (70 mg, 64%) and **21c** (118 mg, 96%) as white solids.

1,2-Dipalmitoyl-3-*O*-(*N*-acetyl-3',4',6'-tri-*O*-benzyl-2'-amino-2'-deoxy-*α*-D-glucopyranosyl)-*sn*-glycerol (**21a**)

^{1}H-NMR (600 MHz, $CDCl_3$): δ 7.37–7.16 (m, 15H, Ar***H***), 5.47 (d, *J* = 9.6 Hz, 1H, -N***H***CO), 5.22–5.18 (m, 1H, $\boldsymbol{H_{sn-2}}$), 4.83 (d, *J* = 11.5 Hz, 1H, PhC***H***), 4.80 (d, *J* = 10.8 Hz, 1H, PhC***H***), 4.77 (d, *J* = 3.6 Hz, 1H, ***H-1***), 4.65 (d, *J* = 11.5 Hz, 1H, PhC***H***), 4.62 (d, *J* = 12.1 Hz, 1H, PhC***H***), 4.51 (d, *J* = 11.7 Hz, 2H, PhC***H*** × 2), 4.39 (dd, *J* = 11.8, 4.5 Hz, 1H, $\boldsymbol{H_{sn-1a}}$), 4.27 (td, *J* = 10.2, 3.7 Hz, 1H, ***H-2***), 4.03 (dd, *J* = 11.8, 5.9 Hz, 1H, $\boldsymbol{H_{sn-1b}}$), 3.76 (dd, *J* = 11.2, 4.4 Hz, 1H, $\boldsymbol{H_{sn-3a}}$), 3.77–3.71 (m, 3H, ***H-5, H-6*** × 2), 3.70–3.65 (m,

2H, ***H-3***, ***H-4***), 3.53 (dd, *J* = 11.2, 6.0 Hz, 1H, $H_{sn\text{-}3b}$), 2.31–2.26 (m, 4H, 2 × CO–CH_2), 1.85 (s, 3H, COCH_3), 1.63–1.58 (m, 4H, 2 × CO–CH_2–CH_2), 1.32–1.25 (m, 48H, 2 × CH_2–CH_2–(CH_2)$_{12}$–CH_3), 0.88 (t, *J* = 7.0 Hz, 6H, 2 × CH_3); LR-ESI-MS *m*/*z* calcd. for $C_{64}H_{100}NO_{10}$ $[M + H]^+$ 1042.7, found 1042.8.

1,2-Dipalmitoyl-3-*O*-(*N*-hexanoyl-3',4',6'-tri-*O*-benzyl-2'-amino-2'-deoxy-*α*-D-glucopyranosyl)-*sn*-glycerol (**21b**)

^{1}H-NMR (500 MHz, $CDCl_3$): δ 7.35–7.14 (m, 15H, Ar*H*), 5.42 (d, *J* = 9.2 Hz, 1H, –N*H*CO), 5.21–5.16 (m, 1H, $H_{sn\text{-}2}$), 4.81 (d, *J* = 11.5 Hz, 1H, PhC*H*), 4.78 (d, *J* = 3.5 Hz, 1H, ***H-1***),4.77 (d, *J* = 10.8 Hz, 1H, PhC*H*), 4.66 (d, *J* = 11.5 Hz, 1H, PhC*H*), 4.62 (d, *J* = 12.2 Hz, 1H, PhC*H*), 4.53–4.49 (m, 2H, PhC*H* × 2), 4.37 (dd, *J* = 11.8, 4.2 Hz, 1H, $H_{sn\text{-}1a}$), 4.29 (td, *J* = 9.6,3.2 Hz, 1H, ***H-2***), 4.05 (dd, *J* = 11.8, 5.9 Hz, 1H, $H_{sn\text{-}1b}$), 3.76 (dd, *J* = 11.0, 4.4 Hz, 1H, $H_{sn\text{-}3a}$), 3.76–3.71 (m, 3H, ***H-5***, ***H-6*** × 2), 3.70–3.64 (m, 2H, ***H-3***, ***H-4***), 3.52 (dd, *J* = 11.0, 5.9 Hz, 1H, $H_{sn\text{-}3b}$), 2.31–2.25 (m, 4H, 2 × CO–CH_2), 2.03 (td, *J* = 7.4, 4.0 Hz, 2H, NHCO–CH_2), 1.60–1.51 (m, 6H, 2 × CO–CH_2–CH_2, NHCO–CH_2–CH_2), 1.33–1.24 (m, 52H, 2 × CO–CH_2–CH_2–(CH_2)$_{12}$–CH_3, NHCO–CH_2–CH_2–(CH_2)$_2$–CH_3), 0.89–0.83 (m, 9H, 3 × CH_3); HR-MALDI-MS *m*/*z* calcd. for $C_{68}H_{107}O_{10}N$ Na $[M + Na]^+$ 1120.7895, found 1120.7786.

1,2-Dipalmitoyl-3-*O*-(*N*-palmitoyl-3',4',6'-tri-*O*-benzyl-2'-amino-2'-deoxy-*α*-*D*-glucopyranosyl)-*sn*-glycerol (**21c**)

^{1}H-NMR (500 MHz, $CDCl_3$): δ 7.34–7.14 (m, 15H, Ar*H*), 5.42 (d, *J* = 9.2 Hz, 1H, –N*H*CO), 5.22–5.17 (m, 1H, $H_{sn\text{-}2}$), 4.81 (d, *J* = 11.5 Hz, 1H, PhC*H*), 4.78 (d, *J* = 3.9 Hz, 1H, ***H-1***), 4.77 (d, *J* = 10.3 Hz, 1H, PhC*H*), 4.66 (d, *J* = 11.5 Hz, 1H, PhC*H*), 4.62 (d, *J* = 12.1 Hz, 1H, PhC*H*), 4.53–4.49 (m, 2H, PhC*H* × 2), 4.37 (dd, *J* = 11.8, 4.2 Hz, 1H, $H_{sn\text{-}1a}$), 4.29 (td, *J* = 9.7,3.5 Hz, 1H, ***H-2***), 4.05 (dd, *J* = 11.8, 5.8 Hz, 1H, $H_{sn\text{-}1b}$), 3.75 (dd, *J* = 10.9, 4.3 Hz, 1H, $H_{sn\text{-}3a}$), 3.76–3.71 (m, 3H, ***H-5***, ***H-6*** × 2), 3.70–3.64 (m, 2H, ***H-3***, ***H-4***), 3.52 (dd, *J* = 10.9, 5.9 Hz, 1H, $H_{sn\text{-}3b}$), 2.31–2.25 (m, 4H, 2 × CO–CH_2), 2.07–2.01 (m, 2H, NHCO–CH_2), 1.65–1.52 (m, 6H, 2 × CO–CH_2–CH_2, NHCO–CH_2–CH_2), 1.34–1.24 (m, 72H, 3 × CO–CH_2–CH_2–(CH_2)$_{12}$–CH_3), 0.88 (t, *J* = 6.8 Hz, 9H, 3 × CH_3); HR-MALDI-MS *m*/*z* calcd. for $C_{78}H_{127}O_{10}N$ Na $[M + Na]^+$ 1260.9358, found 1260.9343.

3-*O*-(2',3',4'-tri-*O*-benzyl-6'-*O*-tert-butyl-diphenylsilyl-*α*-D-glucopyranosyl)-1,2-isopropylidene-*sn*-glycerol (**24**)

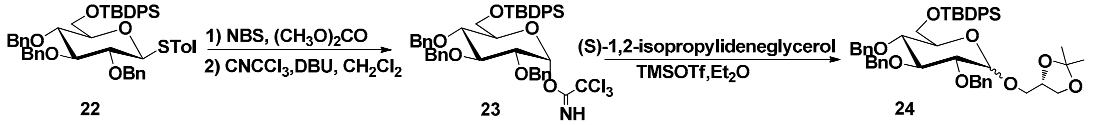

Compound **22** (1.7 g, 2.2 mmol) was treated according to above procedure (synthesis of compound **9**) to give **24** as an oil (1.2 g, 69% for 3 steps). ^{1}H-NMR (600 MHz, $CDCl_3$, *α*/β = 13/1) *α* isomer: δ 7.71–7.66 (m, 4H, Ar*H*), 7.44–7.25 (m, 19H, Ar*H*), 7.16–7.14 (m, 2H, Ar*H*), 4.98 (d, *J* = 10.7 Hz, 1H, PhC*H*), 4.91 (d, *J* = 3.6 Hz, 1H, ***H-1***), 4.89 (d, *J* = 10.8 Hz, 1H, PhC*H*), 4.84–4.79 (m, 2H, PhC*H* × 2), 4.71 (d, *J* = 11.9 Hz, 1H, PhC*H*), 4.61 (d, *J* = 10.8 Hz, 1H, PhC*H*), 4.38–4.34 (m, 1H, $H_{sn\text{-}2}$), 4.06 (dd, *J* = 8.3, 6.5 Hz, 1H, $H_{sn\text{-}3a}$), 3.99 (t, *J* = 9.3 Hz, 1H, ***H-3***), 3.88–3.86 (m, 2H, ***H-6*** × 2), 3.71 (dt, *J* =10.0, 2.9 Hz, ***H-5***), 3.69 (dd, *J* = 8.3, 6.2 Hz, 1H, $H_{sn\text{-}3b}$), 3.65–3.55 (m, 4H, ***H-2***, ***H-4***, $H_{sn\text{-}1a}$, $H_{sn\text{-}1b}$), 1.41 (s, 3H, CH_3), 1.35 (s, 3H, CH_3), 1.05 (s, 9H, CH_3 × 3); LR-ESI-MS *m*/*z* calcd. for $C_{49}H_{58}O_8Si$ Na $[M + Na]^+$ 825.4, found 825.4.

3-*O*-(2',3',4'-tri-*O*-benzyl-*α*-D-glucopyranosyl)-*sn*-glycerol (**25**)

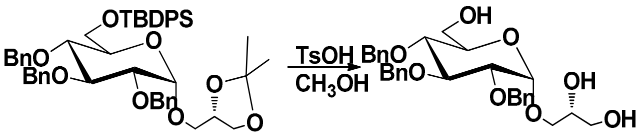

24 **25**

TsOH·H_2O (2.3 mg, 0.01 mmol) was added to a stirred solution of **24** (50 mg, 0.06 mmol) in MeOH (5 mL). After stirring for 8 h at 40 °C, the solution was concentrated and purified by silica

column chromatography (AcOEt-petroleum ether 1:3) to afford the α-anomer **25** (40 mg, 82%) as an oil. ^{1}H-NMR (600 MHz, $CDCl_3$): δ 7.37–7.27 (m, 15H, Ar***H***), 4.94 (d, *J* = 10.9 Hz, 1H, PhC***H***), 4.87 (d, *J* = 11.0 Hz, 1H, PhC***H***), 4.84 (d, *J* = 10.9 Hz, 1H, PhC***H***), 4.79 (d, *J* = 11.8 Hz, 1H, PhC***H***), 4.69 (d, *J* = 3.6 Hz, 1H, ***H-1***), 4.65–4.63 (m, 2H, PhC***H*** × 2), 3.97 (t, *J* = 9.3 Hz, 1H, ***H-3***), 3.86 (dt, *J* = 7.6, 3.9 Hz, 1H, $H_{sn\text{-}2}$), 3.80 (dd, *J* = 10.3, 3.5 Hz, 1H, $H_{sn\text{-}1a}$), 3.77 (dd, *J* = 11.8, 2.3 Hz, 1H, ***H-6a***), 3.73–3.69 (m, 2H, ***H-6b***, ***H-5***), 3.65 (dd, *J* = 11.6, 4.5 Hz, 1H, $H_{sn\text{-}3a}$), 3.61 (dd, *J* = 11.6, 4.5 Hz, 1H, $H_{sn\text{-}3b}$), 3.53–3.48 (m, 2H, ***H-4***, ***H-2***), 3.40 (dd, *J* = 10.3, 6.9 Hz, 1H, $H_{sn\text{-}1b}$); LR-ESI-MS *m*/*z* calcd. for $C_{30}H_{36}O_8$ Na $[M + Na]^+$ 547.2, found 547.2.

1,2-Dipalmitoyl-3-*O*-(6'-*O*-tert-butyl-diphenylsilyl-2',3',4'-tri-*O*-benzyl-α-D-glucopyranosyl)-*sn*-glycerol (**26**)

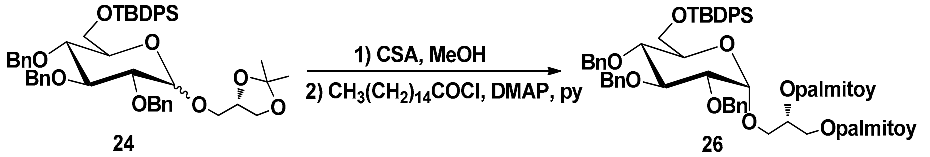

24 **26**

CSA (73 mg, 0.3 mmol) was added to a stirred solution of **24** (2.5 g, 3.1 mmol) in MeOH (30 mL). After stirring for 8 h at r.t, the solution was quenched by Et_3N and concentrated. The residue was purified by silica column chromatography (AcOEt-petroleum ether 1:3) to afford the α-anomer paste. To the solution of the paste in dry pyridine (25 mL), DMAP (57 mg, 0.3 mmol) and palmitoyl chloride (0.5 mL, 1.8 mmol) were added at 80 °C. The reaction mixture was stirred for 3 h and concentrated, then the residue was diluted with CH_2Cl_2 (30 mL) and washed sequentially with 1 M HCl and saturated $NaHCO_3$. The organic phase was dried over Na_2SO_4, filtrated, concentrated. Purification by flash chromatography (AcOEt-petroleum ether 1:10) yielded compound **26** (3.1 g, 82% for 2 steps) as a white solid. H-NMR (600 MHz, $CDCl_3$): δ 7.68–7.64 (m, 4H, Ar***H***), 7.41–7.24 (m, 19H, Ar***H***), 7.16–7.14 (m, 2H, Ar***H***), 5.25–5.21 (m, 1H, $H_{sn\text{-}2}$), 4.95 (d, *J* = 10.8 Hz, 1H, PhC***H***), 4.89 (d, *J* = 10.7 Hz, 1H, PhC***H***), 4.81 (d, *J* = 10.6 Hz, 1H, PhC***H***), 4.80 (d, *J* = 3.6 Hz, 1H, ***H-1***), 4.77 (d, *J* = 11.8 Hz, 1H, PhC***H***), 4.67 (d, *J* = 11.8 Hz, 1H, PhC***H***), 4.62 (d, *J* = 11.0 Hz, 1H, PhC***H***), 4.40 (dd, *J* = 12.0, 3.6 Hz, 1H, $H_{sn\text{-}1a}$), 4.15 (dd, *J* = 12.0, 6.2 Hz, 1H, $H_{sn\text{-}1b}$), 3.97 (t, *J* = 8.0 Hz, 1H, ***H-3***), 3.89–3.82 (m, 2H, ***H-2***, ***H-5***), 3.72 (dd, *J* = 10.7, 5.7 Hz, 1H, $H_{sn\text{-}3a}$), 3.67–3.65 (m, 2H, ***H-6*** × 2), 3.56–3.52 (m, 2H, ***H-4***, $H_{sn\text{-}3b}$), 2.36–2.33 (m, 4H, 2 × CO–CH_2), 1.64–1.60 (m, 4H, 2 × CO–CH_2–CH_2), 1.33–1.20 (m, 48H, 2 × CH_2–CH_2–$(CH_2)_{12}$–CH_3), 1.03 (s, 9H, CH_3 × 3), 0.88 (t, *J* = 7.0 Hz, 6H, CH_3 × 2); LR-ESI-MS *m*/*z* calcd. for $C_{78}H_{114}O_{10}$Si Na $[M + Na]^+$ 1261.8, found 1261.9.

1,2-Dipalmitoyl-3-*O*-(2',3',4'-tri-*O*-benzyl-α-D-glucopyranosyl)-*sn*-glycerol (**27**)

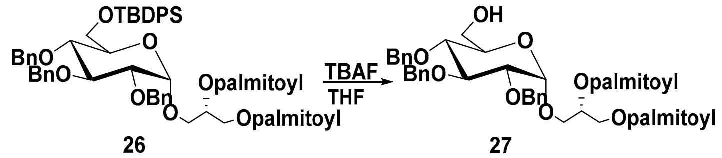

26 **27**

To a solution of **26** (0.5 g, 0.4 mmol) in THF (15 mL), TBAF (0.15 g, 0.5 mmol) was added at 40 °C. The reaction mixture was stirred overnight, and then was concentrated and purified by flash chromatography (AcOEt-petroleum ether 1:6) to afford **27** (0.34 g, 84%) as a white solid. ^{1}H-NMR (600 MHz, $CDCl_3$): δ 7.38–7.28 (m, 15H, Ar***H***), 5.28–5.23 (m, 1H, $H_{sn\text{-}2}$), 4.98 (d, *J* = 10.9 Hz, 1H, PhC***H***), 4.89 (d, *J* = 11.0 Hz, 1H, PhC***H***), 4.83 (d, *J* = 10.9 Hz, 1H, PhC***H***), 4.77 (d, *J* = 12.0 Hz, 1H, PhC***H***), 4.71 (d, *J* = 3.6 Hz, 1H, ***H-1***), 4.66–4.62 (m, 2H, PhC***H*** × 2), 4.42 (dd, *J* = 12.0, 3.6 Hz, 1H, $H_{sn\text{-}1a}$), 4.21 (dd, *J* = 12.0, 6.1 Hz, 1H, $H_{sn\text{-}1b}$), 3.97 (t, *J* = 9.3, 1H, ***H-3***), 3.78–3.72 (m, 2H, ***H-2***, ***H-5***), 3.70–3.64 (m, 2H, $H_{sn\text{-}3a}$, ***H-6a***), 3.57–3.49 (m, 3H, ***H-4***, $H_{sn\text{-}3b}$, ***H-6b***), 2.32–2.28 (m, 4H, 2 × CO–CH_2), 1.64–1.58 (m, 4H, 2 × CO–CH_2–CH_2), 1.32–1.22 (m, 48H, 2 × CH_2–CH_2–$(CH_2)_{12}$–CH_3), 0.89 (t, *J* = 7.0 Hz, 6H, 2 × CH_3); HR-ESI-MS *m*/*z* calcd. for $C_{62}H_{97}O_{10}$ $[M + H]^+$ 1001.7076, found 1001.7078.

1,2-Dipalmitoyl-3-*O*-(2',3',4'-tri-*O*-benzyl-6-iodo-α-D-glucopyranosyl)-*sn*-glycerol (**29**)

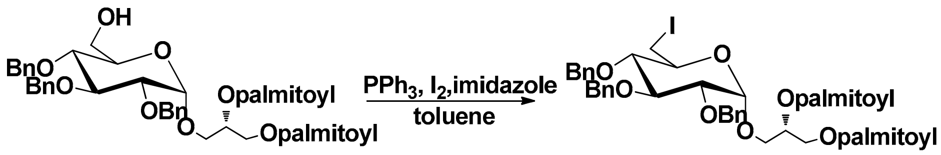

27 **29**

PPh_3 (16 mg, 0.07 mmol) was added to a solution of **27** (60 mg, 0.06 mmol) in toluene (5 mL) under nitrogen atomosphere followed by refluxing for 20 min. The mixture was cooled down to 80 °C and imidazole (11 mg, 0.15 mmol) and I_2 (16 mg, 0.07 mmol) were added. After refluxing for 30 min, the solution was concentrated under reduced pressure. The resulting residue was diluted with CH_2Cl_2 (20 mL) and washed with a saturated $Na_2S_2O_3$ solution (20 mL) and water (20 mL). The organic layer was dried on Na_2SO_4 and evaporated to dryness. Purification by column chromatography (petroleum ether/AcOEt 9:1) gave **29** (46 mg, 70%) as a white solid. ^{1}H-NMR (600 MHz, $CDCl_3$): δ 7.36–7.27 (m, 15H, Ar***H***), 5.28–5.24 (m, 1H, $\boldsymbol{H}_{sn\text{-}2}$), 4.97 (d, *J* = 10.7 Hz, 1H, PhC***H***), 4.94 (d, *J* = 10.9 Hz, 1H, PhC***H***), 4.79 (d, *J* = 10.8 Hz, 1H, PhC***H***), 4.75 (d, *J* = 12.0 Hz,1H, PhC***H***), 4.74 (d, *J* = 3.6 Hz, 1H, ***H-1***), 4.69 (d, *J* = 10.9 Hz, 1H, PhC***H***), 4.62 (d, *J* = 12.0 Hz,1H, PhC***H***),4.41 (dd, *J* = 12.0, 3.7 Hz, 1H, $\boldsymbol{H}_{sn\text{-}1a}$), 4.20 (dd, *J* = 11.9, 6.1 Hz, 1H, $\boldsymbol{H}_{sn\text{-}1b}$), 3.98 (t, *J* = 9.2 Hz, 1H, ***H-3***), 3.84 (dd, *J* = 10.7, 5.3 Hz, 1H, $\boldsymbol{H}_{sn\text{-}3a}$), 3.56–3.51 (m, 2H, ***H-2***, $\boldsymbol{H}_{sn\text{-}3b}$), 3.46–3.42 (m, 2H, ***H-6*** × 2), 3.34 (t, *J* = 9.0 Hz, 1H, ***H-4***), 3.28–3.27 (m, 1H, ***H-5***), 2.32–2.28 (m, 4H, $C\boldsymbol{H}_2$ × 2), 1.62–1.58 (m, 4H, $C\boldsymbol{H}_2$ × 2), 1.31-1.24 (m, 48H, $(C\boldsymbol{H}_2)_{12}$ × 2), 0.88 (t, *J* = 7.1 Hz, 6H, $C\boldsymbol{H}_3$ × 2); LR-ESI-MS calcd. for $C_{62}H_{95}IO_9$ Na $[M + Na]^+$ 1133.6, found 1133.6.

1,2-Dipalmitoyl-3-*O*-(2',3',4'-tri-*O*-benzyl-*α*-D-glucosyluronate)-*sn*-glycerol (30)

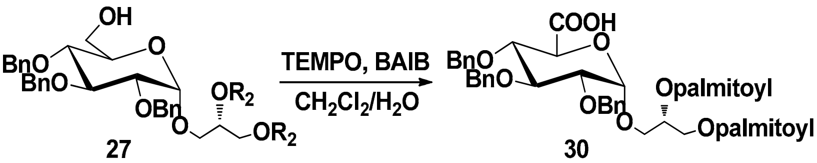

27 **30**

TEMPO (3 mg, 0.02 mmol) and BAIB (80 mg, 0.25 mmol) were added to a mixture of **27** (110 mg, 0.1 mmol) in CH_2Cl_2 (2 mL) and H_2O (1 mL). The emulsion was vigorously stirred overnight at room temperature and the reaction was quenched with saturated $Na_2S_2O_3$ solution. After extraction of the aqueous layer with CH_2Cl_2, the organic layer was washed with sat. $NaHCO_3$ and brine, dried over Na_2SO_4 and evaporated. The residue was purified by column chromatography (petroleum ether/AcOEt 3:1) to afford compound **30** (0.10 g, 92%) as a yellow oil. ^{1}H-NMR (600 MHz, $CDCl_3$): δ 7.34–7.22 (m, 15H, Ar***H***), 5.26–5.22 (m, 1H, $\boldsymbol{H}_{sn\text{-}2}$), 4.95 (d, *J* = 10.8 Hz, 1H, PhC***H***), 4.83 (d, *J* = 10.6 Hz, 1H, PhC***H***), 4.81 (d, *J* = 10.9 Hz, 1H, PhC***H***), 4.76 (d, *J* = 12.0 Hz, 1H, PhC***H***), 4.74 (d, *J* = 3.5 Hz, 1H, ***H-1***), 4.64 (d, *J* = 10.7 Hz,1H, PhC***H***), 4.60 (d, *J* = 12.0 Hz, 1H, PhC***H***), 4.40 (dd, *J* = 12.0, 3.7 Hz, 1H, $\boldsymbol{H}_{sn\text{-}1a}$), 4.25 (d, *J* = 10.1 Hz, 1H, ***H-5***), 4.22 (dd, *J* = 12.0, 6.0 Hz, 1H, $\boldsymbol{H}_{sn\text{-}1b}$), 3.97 (t, *J* = 9.3 Hz, 1H, ***H-3***), 3.78 (dd, *J* = 10.8, 5.3 Hz, 1H, $\boldsymbol{H}_{sn\text{-}3a}$), 3.70 (dd, *J* = 9.9, 9.1 Hz, 1H, ***H-4***), 3.57–3.54 (m, 2H, ***H-2***, $\boldsymbol{H}_{sn\text{-}3b}$), 2.32–2.28 (m, 4H, 2 × CO–$C\boldsymbol{H}_2$), 1.61–1.57 (m, 4H, 2 × CO–CH_2–$C\boldsymbol{H}_2$), 1.31–1.23 (m, 48H, 2 × CH_2–CH_2–$(C\boldsymbol{H}_2)_{12}$–CH_3),0.88 (t, *J* = 7.0 Hz, 6H, 2 × $C\boldsymbol{H}_3$); LR-ESI-MS *m*/*z* calcd. for $C_{62}H_{93}O_{11}$ $[M - H]^-$ 1013.7, found 1013.9.

1,2-Dipalmitoyl-3-*O*-(2',3',4'-tri-*O*-benzyl-6'-*O*-sulfo-*α*-D-glucopyranoside)-*sn*-glycerol (**31**)

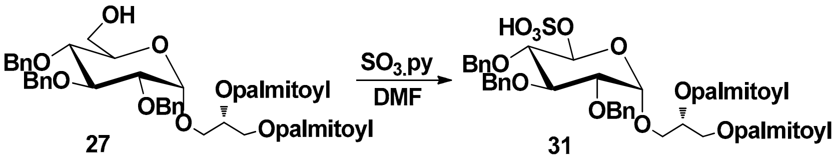

27 **31**

SO_3·py (0.21 g, 1.3 mmol) was added to a solution of **27** (0.13 g, 0.1 mmol) in DMF (8 mL) under nitrogen. After strring for 1 h, the solution was concentrated under reduced pressure. The resulting residue was diluted with CH_2Cl_2 (30 mL) and washed with sat. $NaHCO_3$ and water. The organic layer was dried on Na_2SO_4 and evaporated to dryness. Purification by column chromatography (CH_2Cl_2/CH_3OH 10:1) afforded **31** (0.12 g, 86%) as a colorless oil. ^{1}H-NMR (600 MHz, $CDCl_3$): δ 7.28–7.13 (m, 15H, Ar***H***), 5.23–5.27 (m, 1H, $\boldsymbol{H}_{sn\text{-}2}$), 4.89 (d, *J* = 2.9 Hz, 1H, ***H-1***),4.87 (d, *J* = 11.2 Hz, 1H, PhC***H***),

4.76 (d, *J* = 10.7 Hz, 1H, PhC***H***), 4.73 (d, *J* = 11.1 Hz, 1H, PhC***H***), 4.64 (d, *J* = 10.5 Hz, 1H, PhC***H***), 4.57 (d, *J* = 12.1 Hz, 1H, PhC***H***), 4.54 (d, *J* = 11.8 Hz, 1H, PhC***H***), 4.33–4.21 (m, 3H, $\boldsymbol{H_{sn\text{-}1a}}$, ***H-6*** × 2), 4.15 (dd, *J* = 11.9, 6.8 Hz, 1H, $\boldsymbol{H_{sn\text{-}1b}}$), 3.88 (t, *J* = 9.2 Hz, 1H, ***H-3***), 3.84–3.82 (m, 1H, $\boldsymbol{H_{sn\text{-}3a}}$), 3.78 (dd, *J* = 11.0, 3.6 Hz, 1H, $\boldsymbol{H_{sn\text{-}3b}}$), 3.57–3.47 (m, 3H, ***H-5***, ***H-2***, ***H-4***), 2.32–2.28 (m, 4H, 2 × CO–C$\boldsymbol{H}_2$), 1.61–1.57 (m, 4H, 2 × CO–CH_2–C$\boldsymbol{H}_2$), 1.31–1.23 (m, 48H, 2 × CH_2–CH_2–(C$\boldsymbol{H}_2$)$_{12}$–CH_3), 0.88 (t, *J* = 7.0 Hz, 6H, C$\boldsymbol{H}_3$ × 2); LR-ESI-MS *m/z* calcd. for $C_{62}H_{95}O_{13}S$ $[M - H]^-$ 1079.6, found 1079.9.

1,2-Dipalmitoyl-3-*O*-[2'',3'',4'',6''-tetra-*O*-benzyl-α-D-galactopyranosyl-(1''→6')-2',3',4'-tri-*O*-benzyl-α-D-glucopyranosyl]-*sn*-glycerol (**32**)

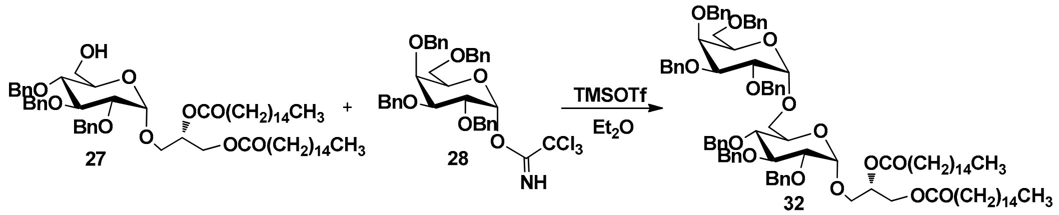

To a solution of compound **27** (0.10 g, 0.10 mmol), **28** [3] (82 mg, 0.12 mmol) and 4 Å molecular sieves in dry CH_2Cl_2 (12 mL) at 0 °C under nitrogen was added TMSOTf (4 μL, 0.02 mmol). The mixture was stirred for 1 h at 0 °C and then neutralized by Et_3N and filtered. The filtrate was concentrated to give a residue that was purified by column chromatography (petroleum ether-AcOEt, 8:1) affording compound **32** as a colorless oil (114 mg, 75%). ^{1}H-NMR (600 MHz, $CDCl_3$): δ 7.35–7.19 (m, 35H, Ar***H***), 5.23–5.19 (m, 1H, $\boldsymbol{H_{sn\text{-}2}}$), 5.03 (d, *J* = 3.7 Hz, 1H, ***H-1''***), 4.94–4.90 (m, 2H, PhC***H*** × 2), 4.85 (d, *J* = 11.0 Hz, 1H, PhC***H***), 4.81–4.77 (m, 2H, PhC***H*** × 2), 4.72–4.70 (m, 3H, PhC***H*** × 3), 4.67 (d, *J* = 3.4 Hz, 1H, ***H-1'***), 4.62–4.60 (m, 2H, PhC***H*** × 2), 4.55 (d, *J* = 11.5 Hz, 1H, PhC***H***), 4.48 (d, *J* = 12.0 Hz, 1H, PhC***H***), 4.43 (d, *J* = 11.7 Hz, 1H, PhC***H***), 4.38 (dd, *J* = 11.9, 3.3 Hz, 1H, $\boldsymbol{H_{sn\text{-}1a}}$), 4.55 (d, *J* = 12.0 Hz, 1H, PhC***H***), 4.17 (dd, *J* = 11.9, 6.2 Hz, 1H, $\boldsymbol{H_{sn\text{-}1b}}$), 4.03 (dd, *J* = 9.8, 3.7 Hz, 1H, ***H-2''***), 3.94–3.84 (m, 4H, ***H-3'***, ***H-3''***, ***H-4''***, ***H-5''***), 3.74–3.68 (m, 4H, ***H-6'*** × 2, ***H-5'***, $\boldsymbol{H_{sn\text{-}3a}}$), 3.55–3.47 (m, 2H, ***H-6''*** × 2, ***H-4'***, $\boldsymbol{H_{sn\text{-}3b}}$), 3.37 (dd, *J* = 9.5, 3.4 Hz, 1H, ***H-2'***), 2.36–2.23 (m, 4H, COC$\boldsymbol{H}_2$ × 2), 1.67–1.56 (m, 4H, O=CCH_2C$\boldsymbol{H}_2$ × 2), 1.30–1.23 (m, 48H, (C$\boldsymbol{H}_2$)$_{12}$ × 2), 0.88 (t, *J* = 6.9 Hz, 6H, C$\boldsymbol{H}_3$ × 2); LR-MALDI-MS *m/z* calcd. for $C_{96}H_{130}O_{15}$ Na $[M + Na]^+$ 1545.9, found 1545.7.

Bis(tetradecyl)methyl-6-azide-2,3,4-tri-*O*-benzyl-6-deoxy-α-D-glucopyranoside (**33**)

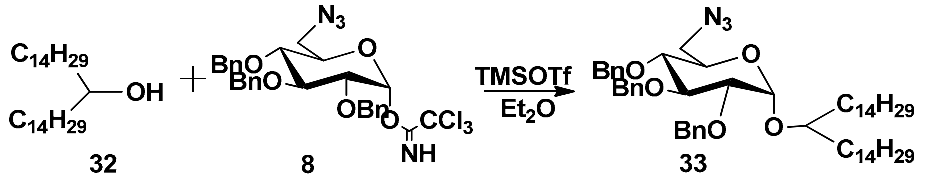

A solution of **32** [4] (0.5 g, 1.2 mmol) in dry Et_2O (35 mL) was added freshly dried powdered 4 Å MS and cooled to 0 °C, then catalytic amount of TMSOTf (35 μL, 1.2 mmol) was added and the mixture was stirred for 20 min at room temperature. The solution of **8** (0.6 g, 0.97 mmol) in dry Et_2O (10 mL) was slowly added and stirred for 30 min. The mixture was quenched by Et_3N and filtered to remove 4 Å MS. The filtrate was concentrated and purified by column chromatography on silica gel (petroleum ether/AcOEt: 15/1) to afford **33** (0.42 g, 49%) as a white solid. ^{1}H-NMR (500 MHz, $CDCl_3$): δ 7.35–7.27 (m, 15H, Ar***H***), 5.00 (d, *J* = 10.8 Hz, 1H, PhC***H***), 4.94 (d, *J* = 3.7 Hz, 1H, ***H-1***), 4.91 (d, *J* = 11.0 Hz, 1H, PhC***H***), 4.81 (d, *J* = 10.8 Hz, 1H, PhC***H***), 4.73 (d, *J* = 11.7 Hz, 1H, PhC***H***), 4.73 (d, *J* = 11.7 Hz, 1H, PhC***H***), 4.59 (d, *J* = 11.0 Hz, 1H, PhC***H***), 3.99 (t, *J* = 9.3 Hz, 1H, ***H-3***), 3.92 (ddd, *J* = 9.7, 5.4, 2.4 Hz, 1H, ***H-5***), 3.59–3.56 (m, 1H, –OC***H***–), 3.53 (dd, *J* = 9.7, 3.8 Hz, 1H, ***H-2***), 3.46–3.40 (m, 2H, ***H-4***, ***H-6a***), 3.35 (dd, *J* = 13.0, 5.4 Hz, 1H, ***H-6b***), 1.53–1.48 (m, 4H, OCH–(C$\boldsymbol{H}_2$)$_2$), 1.27–1.22 (m, 48H, (C$\boldsymbol{H}_2$)$_{12}$ × 2), 0.88 (t, *J* = 7.0 Hz, 6H, C$\boldsymbol{H}_3$ × 2); HR-MALDI-MS *m/z* calcd. for $C_{56}H_{87}O_5N_3$ Na 904.6543 $[M + Na]^+$, found 904.6555.

Bis(tetradecyl)methyl-6-amino-2,3,4-tri-*O*-benzyl-6-deoxy-α-D-glucopyranoside (**34**)

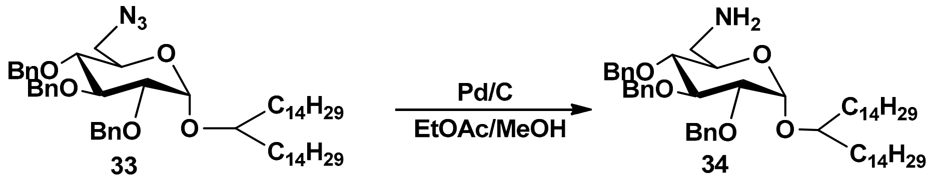

To a solution of **33** (60 mg, 0.07 mmol) in 4 mL AcOEt/MeOH (1:1) was treated with 10% palladium (30 mg) and stirred at r.t. under hydrogen atmosphere for 2 h. After filtration the solvent was evaporated and the residue was purified by silica column chromatography (CH_2Cl_2–MeOH 15:1) to afford *α*-anomer **34** (51 mg, 88%) as a yellow oil. ^{1}H-NMR (500 MHz, $CDCl_3$): δ 7.35–7.29 (m, 15H, Ar***H***), 5.00 (d, *J* = 10.8 Hz, 1H, PhC***H***), 4.88 (d, *J* = 3.7 Hz, 1H, ***H-1***), 4.89 (d, *J* = 11.0 Hz, 1H, PhC***H***), 4.82 (d, *J* = 10.8 Hz, 1H, PhC***H***), 4.73 (d, *J* = 11.7 Hz, 1H, PhC***H***), 4.68–4.63 (m, 2H, PhC***H*** × 2), 4.00 (t, *J* = 9.3 Hz, 1H, ***H-3***), 3.74–3.69 (m, 3H, ***H-5***, –N$\boldsymbol{H}_2$), 3.56–3.53 (m, 1H, OC***H***), 3.48 (dd, *J* = 9.8, 3.7 Hz, 1H, ***H-2***), 3.37 (t, *J* = 9.4 Hz, 1H, ***H-4***), 2.97 (dd, *J* = 13.4, 2.6 Hz, 1H, ***H-6a***), 3.35 (dd, *J* = 13.4, 5.5 Hz, 1H, ***H-6b***), 1.51–1.47 (m, 4H, –OCH–$(C\boldsymbol{H}_2)_2$), 1.27–1.22 (m, 48H, –$(C\boldsymbol{H}_2)_{12}$ × 2), 0.88 (t, *J* = 6.9 Hz, 6H, $C\boldsymbol{H}_3$ × 2); LR-ESI-MS *m*/*z* calcd. for $C_{56}H_{90}NO_5$ $[M + H]^+$ 856.7, found 856.6.

General procedures for **35a**–**35c**

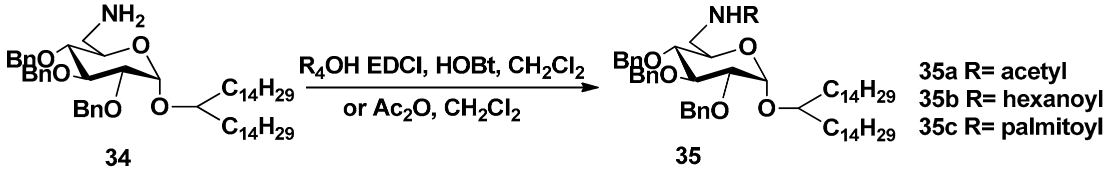

To a solution of compound **34** (80 mg, 0.09 mmol) in CH_2Cl_2 (5 mL) was added Ac_2O (20 μL, 0.2 mmol) and DMAP (2 mg, 0.02 mmol) at 0 °C. After stirring for 1 h, the mixture was washed with saturated $NaHCO_3$ and brine. The organic layer was dried over Na_2SO_4 and concentrated. The residue was chromatographed (AcOEt-petroleum ether 1:4) to afford **35a** (70 mg, 87%) as a white solid.

To a solution of compound **34** (80 mg, 0.09 mmol) in CH_2Cl_2 (5 mL) were sequentially added EDCI (21 mg, 0.11 mmol), HOBt (15 mg, 0.11 mmol) and corresponding fatty acids (hexadecanoic acid, or palmitic acid, 1.2 eq) at 0 °C. After stirring for 1 h, the mixture was diluted with saturated $NaHCO_3$ and brine. The organic layer was dried over Na_2SO_4 and concentrated. The residue was chromatographed (AcOEt-petroleum ether 1:8) to afford **35b** (50 mg, 58%) and **35c** (85 mg, 86%) respectively as white solids.

Bis(tetradecyl)methyl-*N*-acetyl-2,3,4-tri-*O*-benzyl-6-amino-6-deoxy-*α*-D-glucopyranoside (**35a**)

^{1}H-NMR (500 MHz, $CDCl_3$): δ 7.38–7.28 (m, 15H, Ar***H***), 5.57 (dd, *J* = 6.2, 3.9 Hz, 1H, –N***H***), 4.99 (d, *J* = 10.8 Hz, 1H, PhC***H***), 4.87 (d, *J* = 3.9 Hz, 1H, ***H-1***), 4.86–4.82 (m, 2H, PhC***H*** × 2), 4.74 (d, *J* = 12.0 Hz, 1H, PhC***H***),4.68 (d, *J* = 12.0 Hz, 1H, PhC***H***), 4.63 (d, *J* = 10.5 Hz, 1H, PhC***H***), 4.00 (t, *J* = 9.3 Hz, 1H, ***H-3***), 3.84–3.80 (m, 1H, ***H-6a***), 3.75–3.70 (m, 1H, ***H-5***), 3.53–3.49 (m, 1H OC***H***), 3.47 (dd, *J* = 9.8, 3.7 Hz, 1H, ***H-2***), 3.35 (dt, *J* = 13.9, 3.7 Hz, 1H, ***H-6b***), 3.28 (t, *J* = 9.3 Hz, 1H, ***H-4***), 1.92 (s, 3H, COC$\boldsymbol{H}_3$), 1.51–1.47 (m, 4H, OCH–$(C\boldsymbol{H}_2)_2$), 1.35–1.22 (m, 48H, $(C\boldsymbol{H}_2)_{12}$ × 2), 0.88 (t, *J* = 6.9 Hz, 6H, $C\boldsymbol{H}_3$ × 2); HR-MALDI-MS *m*/*z* calcd. for $C_{58}H_{91}O_6N$ Na $[M + Na]^+$ 920.6739, found 920.6748.

Bis(tetradecyl)methyl-*N*-hexanoyl-2,3,4-tri-*O*-benzyl-6-amino-6-deoxy-*α*-D-glucopyranoside (**35b**)

^{1}H-NMR (500 MHz, $CDCl_3$): δ 7.38-7.27 (m, 15H, Ar***H***), 5.58 (dd, *J* = 6.5, 4.2 Hz, 1H, –N***H***), 4.99 (d, *J* = 10.8 Hz, 1H, PhC***H***), 4.87 (d, *J* = 3.7 Hz, 1H, ***H-1***), 4.84 (d, *J* = 10.0 Hz, 2H, PhC***H*** × 2), 4.74 (d, *J* = 11.8 Hz, 1H, PhC***H***), 4.68 (d, *J* = 11.8 Hz, 1H, PhC***H***), 4.63 (d, *J* = 10.5 Hz, 1H, PhC***H***), 4.00 (t, *J* = 9.3 Hz, 1H, ***H-3***), 3.84–3.80 (m, 1H, ***H-6a***), 3.78–3.72 (m, 1H, ***H-5***), 3.54–3.50 (m, 1H OC***H***), 3.46 (dd, *J* = 9.8, 3.4 Hz, 1H, ***H-2***), 3.37 (dt, *J* = 13.7, 3.7 Hz, 1H, ***H-6b***), 3.28 (t, *J* = 9.4 Hz, 1H, ***H-4***), 2.11 (t, *J* = 7.7 Hz, 2H, COC$\boldsymbol{H}_2$), 1.63–1.58 (m, 4H, OCH–$(C\boldsymbol{H}_2)_2$), 1.50–1.47 (m, 2H, $COCH_2C\boldsymbol{H}_2$), 1.34–1.25 (m, 52H, $(C\boldsymbol{H}_2)_{12}$ × 2, $COCH_2CH_2(C\boldsymbol{H}_2)_2$), 0.90–0.86 (m, 9H, $C\boldsymbol{H}_3$ × 3); HR-MALDI-MS *m*/*z* calcd. for $C_{62}H_{99}O_6N$ Na $[M + Na]^+$ 976.7365, found 976.7379.

Bis(tetradecyl)methyl-*N*-palmitoyl-2,3,4-tri-*O*-benzyl-6-amino-6-deoxy-α-D-glucopyranoside (**35c**)

^{1}H-NMR (500 MHz, $CDCl_3$): δ 7.37–7.27 (m, 15H, Ar***H***), 5.57 (dd, *J* = 6.9, 4.0 Hz, 1H, –N***H***), 4.98 (d, *J* = 10.8 Hz, 1H, PhC***H***), 4.87 (d, *J* = 3.7 Hz, 1H, ***H-1***), 4.88–4.82 (m, 2H, PhC***H*** × 2), 4.74 (d, *J* = 11.8Hz, 1H, PhC***H***), 4.68 (d, *J* = 11.8 Hz, 1H, PhC***H***), 4.63 (d, *J* = 10.3 Hz, 1H, PhC***H***), 3.99 (t, *J* = 9.2 Hz, 1H, ***H-3***), 3.84–3.79 (m, 1H, ***H-6a***), 3.76–3.71 (m, 1H, ***H-5***), 3.53–3.49 (m, 1H OC***H***), 3.46 (dd, *J* = 9.7, 3.6 Hz, 1H, ***H-2***), 3.37 (dt, *J* = 13.8, 3.9 Hz, 1H, ***H-6b***), 3.28 (t, *J* = 9.4 Hz, 1H, ***H-4***), 2.11 (t, *J* = 7.6 Hz, 2H, $COC\boldsymbol{H}_2$), 1.58–1.47 (m, 6H, $COCH_2C\boldsymbol{H}_2$, $OCH(C\boldsymbol{H}_2)_2$), 1.35–1.22 (m, 72H, $COCH_2CH_2(C\boldsymbol{H}_2)_{12}$, $(C\boldsymbol{H}_2)_{12}$ × 2), 0.88 (t, *J* = 6.9 Hz, 9H, $C\boldsymbol{H}_3$ × 3); HR-MALDI-MS *m/z* calcd. for $C_{72}H_{119}O_6N$ Na [M + Na]$^+$ 1116.8930, found 1116.8927.

References

1 Vasella, A.; Witzig, C.; Chiara, J.L.; Martin-Lomas, M. Convenient Synthesis of 2-Azido-2-deoxy-aldoses by Diazo Transfer. *HeIv. Chim. Acta* **2004**, *74*, 2073–2077.

2 Goddard-Borger, E.D.; Stick, R.V. An efficient, inexpensive, and shelf-stable diazotransfer reagent: Imidazole-1-sulfonyl azide hydrochloride. *Org. Lett.* **2007**, *9*, 3797–3800.

3 Kim, S.; Song, S.; Lee, T.; Jung, S.; Kim, D. Practical synthesis of KRN7000 from phytosphingosine. *Synthesis* **2004**, *6*, 847–850.

4 Roosjen, A.; Šmisterová, J.; Driessen, C.; Anders, J.T.; Wagenaar, A.; Hoekstra, D.; Hulst, R.; Engberts, J.B. Synthesis and characteristics of biodegradable pyridinium amphiphiles used for *in vitro* DNA delivery. *Eur. J. Org. Chem.* **2002**, *2002*, 1271–1277.
